# Integration of Data-Dependent Acquisition (DDA) and Data-Independent High-Definition MS^E^ (HDMS^E^) for the Comprehensive Profiling and Characterization of Multicomponents from *Panax japonicus* by UHPLC/IM-QTOF-MS

**DOI:** 10.3390/molecules24152708

**Published:** 2019-07-25

**Authors:** Chunxia Zhang, Tiantian Zuo, Xiaoyan Wang, Hongda Wang, Ying Hu, Zheng Li, Weiwei Li, Li Jia, Yuexin Qian, Wenzhi Yang, Heshui Yu

**Affiliations:** 1 Tianjin State Key Laboratory of Modern Chinese Medicine, Tianjin University of Traditional Chinese Medicine, Tianjin 300193, China; 2 Tianjin Key Laboratory of TCM Chemistry and Analysis, Tianjin University of Traditional Chinese Medicine, Tianjin 300193, China; 3 College of Pharmaceutical Engineering of Traditional Chinese Medicine, Tianjin University of Traditional Chinese Medicine, Tianjin 300193, China

**Keywords:** *Panax japonicus*, multicomponent characterization, UHPLC/IM-QTOF-MS, DDA, high-definition MS^E^

## Abstract

The complexity of herbal matrix necessitates the development of powerful analytical strategies to enable comprehensive multicomponent characterization. In this work, targeting the multicomponents from *Panax japonicus* C.A. Meyer, both data dependent acquisition (DDA) and data-independent high-definition MS^E^ (HDMS^E^) in the negative electrospray ionization mode were used to extend the coverage of untargeted metabolites characterization by ultra-high-performance liquid chromatography (UHPLC) coupled to a Vion^TM^ IM-QTOF (ion-mobility/quadrupole time-of-flight) high-resolution mass spectrometer. Efficient chromatographic separation was achieved by using a BEH Shield RP18 column. Optimized mass-dependent ramp collision energy of DDA enabled more balanced MS/MS fragmentation for mono- to penta-glycosidic ginsenosides. An in-house ginsenoside database containing 504 known ginsenosides and 60 reference compounds was established and incorporated into UNIFI^TM^, by which efficient and automated peak annotation was accomplished. By streamlined data processing workflows, we could identify or tentatively characterize 178 saponins from *P. japonicus*, of which 75 may have not been isolated from the *Panax* genus. Amongst them, 168 ginsenosides were characterized based on the DDA data, while 10 ones were newly identified from the HDMS^E^ data, which indicated their complementary role. Conclusively, the in-depth deconvolution and characterization of multicomponents from *P. japonicus* were achieved, and the approaches we developed can be an example for comprehensive chemical basis elucidation of traditional Chinese medicine (TCM).

## 1. Introduction

A key segment involved in the modernization research of traditional Chinese medicine (TCM) is the clarification of chemical compositions [1]. The development of powerful analytical technologies capable of the comprehensive deconvolution of plant metabolites has been a hot topic in the field of analytical chemistry. To date, profiling and characterization of the metabolites from plants or biosamples are preferably performed by tandem mass spectrometry (MS/MS or MS^n^) in an untargeted mode coupled to chromatographic separation [2]. Data-dependent acquisition (DDA) is the most commonly used strategy in untargeted metabolites characterization without the necessity of prior knowledge, by which the top N most intense precursor ions recorded in MS^1^ are selected one at a time for fragmentation by collision-induced dissociation (CID) or high-energy collision-induced dissociation (HCD) [3,4,5]. However, facing the complex composition of ion species, the coverage facilitated by DDA may be restricted since repeatable or useless fragmentation information associated with diverse adducts, dimers, in-source decay fragments, and even doubly charged ions, etc. can be acquired. Data-independent acquisition (DIA) by allowing the fragmentation of all ions within a mass window (Sequential Window Acquisition for All Theoretical Spectra (SWATH) for AB SCIEX (Framingham, MA, USA) [6,7]) or full *m*/*z* range (MS^E^ for Waters (Manchester, UK) [8,9]; All Ions Fragmentation (AIF) for Thermo Fisher Scientific (San Jose, CA, USA) [10,11]; All Ions MS/MS for Agilent (Santa Clara, CA, USA) [12], etc.) can ensure a complete coverage of all precursors [13], but precursor-product ion matching is inevitable prior to interpreting the DIA data, which can be performed either by commercial software or an in-house developed algorithm [14,15]. On occasion, the matching results may suffer from false positives from the adducts or in-source decay fragments. Therefore, we propose to integrate DDA and DIA in an experiment by taking advantage of their merits to generate complementary information supporting comprehensive, untargeted characterization of plant metabolites. In addition, ion mobility facilitates an additional dimension of separation of ions (based on the difference of charge state, size, and shape) which is orthogonal to chromatographic retention and MS. The hybrid ion mobility/quadrupole time-of-flight mass spectrometry (IM/QTOF-MS; such as the commercial SYNAPT series and Vion IM-QTOF from Waters, 6560 QTOF from Agilent) coupled to liquid chromatography (LC) can enable three-dimensional separations providing four dimensions of information related to molecules (t_R_, drift time or collision cross section (CCS), MS^1^, and MS^2^) [16,17]. IM-derived CCS can act as a stable parameter useful for more reliable structural elucidation of a metabolite [18]. 

The *Panax* species has been well-known for the remarkable tonifying effects to humans, among which *P. ginseng* C.A. Meyer (Asian ginseng), *P. quinquefolius* L. (American ginseng), *P. notoginseng* (Burk.) F.H. Chen (Sanchi ginseng), *P. japonicus* C.A. Meyer (Japanese ginseng), and *P. vietnamensis* Ha and Grushv. (Vietnamese ginseng) are globally popular [19]. According to previous phytochemical researches, various primary and secondary metabolites, such as the saponins, polysaccharides, flavonoids, amino acids, organic acids, and sterols, etc., have been isolated from various *Panax* species. The contained saponins, also known as ginsenosides, represent a class of specific and biologically active components closely associated with the therapeutic effects of ginseng on the cardiovascular system and immune system [20,21]. The saponins isolated from the *Panax* genus, according to our previous review [19], have unique structure features: 1) having a sapogenin of tetracyclic dammarane (protopanaxadiol (PPD), protopanaxatriol (PPT), or their derivatives) or pentacyclic oleanolic acid (OA); 2) characteristic sugars including glucose (Glc), glucuronic acid (GlurA), rhamnose (Rha), xylose (Xyl), and arabinose (Ara) in either a furan or pyran form; and 3) various acylation forms such as acetyl, malonyl, butenyl, and octenyl, etc. These structure features of ginsenosides can favor the rapid characterization of ginsenosides from various ginseng drugs by liquid chromatography/mass spectrometry [22].

*P. japonicus* C.A. Meyer (the rhizome) is used as a traditional herbal medicine, Panacis Japonici Rhizoma (Zhu-Jie-Shen), officially recorded in Chinese Pharmacopoeia (2015 edition), with the functions of dissipating blood stasis, detumescence, hemostasis, and reinforcing deficiency [23,24]. According to a review article of Reference [25], at least 111 saponins have been isolated from this species, which can be classified into the PPD, PPT, OT (octillol), OA, UA (ursolic acid), and DD II (dammarenediol II) subtypes. Compared with *P. ginseng*, *P. quinquefolius*, and *P. notoginseng*, one remarkable feature in the composition of *P. japonicus* saponins is the high content of three OA saponins, ginsenoside Ro (C_48_H_76_O_19_), chikusetsusaponins IV (C_47_H_74_O_18_), and IVa (C_42_H_66_O_14_) [26]. We find that, based on a comprehensive literature survey, very few researches try to unveil the complex composition of multicomponents occurring to *P. japonicus*. A recent work reported the characterization of a total of 53 saponins from *P. japonicus* by ultra-high-performance liquid chromatography/quadrupole time-of-flight mass spectrometry (UHPLC/QTOF-MS) analysis, with the aid of 23 reference compounds [27]. Another research could characterize 82 components from *P. japonicus* with 16 reference compounds [28]. Development of a more powerful analytical strategy is crucial for aiming to achieve the in-depth multicomponent characterization for *P. japonicus*, in particular to separate and identify minor components that have been ignored in previous researches.

**Figure 1:**
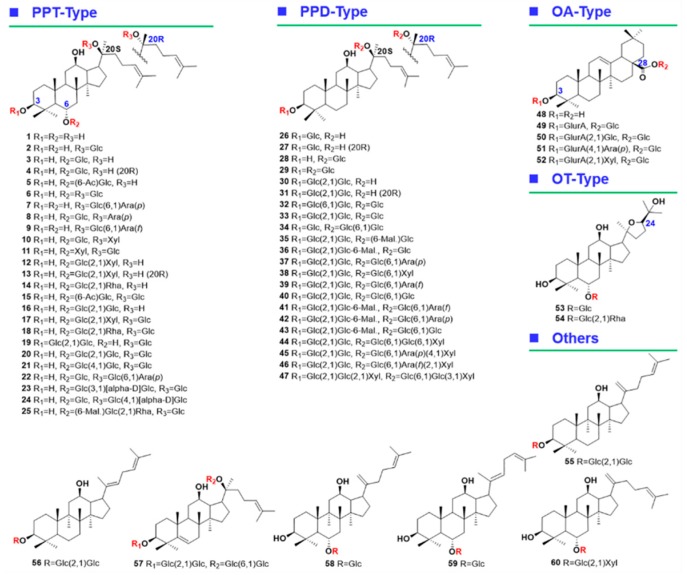
Chemical structures of 60 ginsenoside reference compounds.

The aim of the current work was to integrate two data acquisition approaches, DDA and high-resolution MS^E^ (HDMS^E^), on a powerful Vion^TM^ IM-QTOF hybrid high-resolution mass spectrometer coupled with reversed-phase UHPLC (RP-UHPLC) for the systematic profiling and characterization of multicomponents from *P. japonicus*. For this purpose, numerous efforts had been made: (1) both the chromatography (e.g., stationary phase, mobile phase, column temperature, and gradient elution program) and MS (in particular the ramp collision energy) conditions were carefully optimized; (2) an in-house database recording 504 known ginsenosides and 60 reference compounds (Figure 1) was established to assist the structural elucidation, rendering the results more reliable; and (3) UNIFI^TM^, a versatile data processing platform that had incorporated the ginsenoside database, was utilized to establish streamlined workflows to achieve automated annotation of both the DDA and HDMS^E^ data. Consequently, we could identify or tentatively characterize 178 saponins from *P. japonicus*, and 75 thereof may have not been isolated from the whole *Panax* genus. Complementarity of the DDA and HDMS^E^ data was discussed as well. The current work can demonstrate a potent approach facilitating the comprehensive multicomponent characterization of TCM.

## 2. Results and Discussion

### 2.1. Optimization of the RP-UHPLC Conditions for Well Resolving the Multicomponents from P. japonicus

Aiming to separate and identify as many components as possible from *P. japonicus*, as the first step, the RP-UHPLC conditions were optimized. Key parameters, involving the stationary phase, mobile phase, column temperature, and gradient elution program, had been optimized.

Our previous researches indicated different additives (ammonium acetate, AA; formic acid, FA) in the mobile phase could evidently affect the retention behavior and the selectivity of ginsenosides on RP columns [4,14,22,29,30]. In this work, we firstly compared the influence of AA and FA as the additive of water phase on the resolution of ginsenosides from *P. japonicus* using a BEH Shield RP18 column (2.1 × 100 mm, 1.7 µm) with the same amount of sample loaded (Figure S1). It was very clear much more peaks could be resolved when using 0.1% FA in the mobile phase in contrast to the addition of 3 mM AA in the aqueous phase. It could demonstrate better selectivity of ginsenosides facilitated by FA in the mobile phase than AA. Accordingly, we selected CH_3_CN/0.1% FA-H_2_O as the mobile phase in the following experiments.

**Figure 2:**
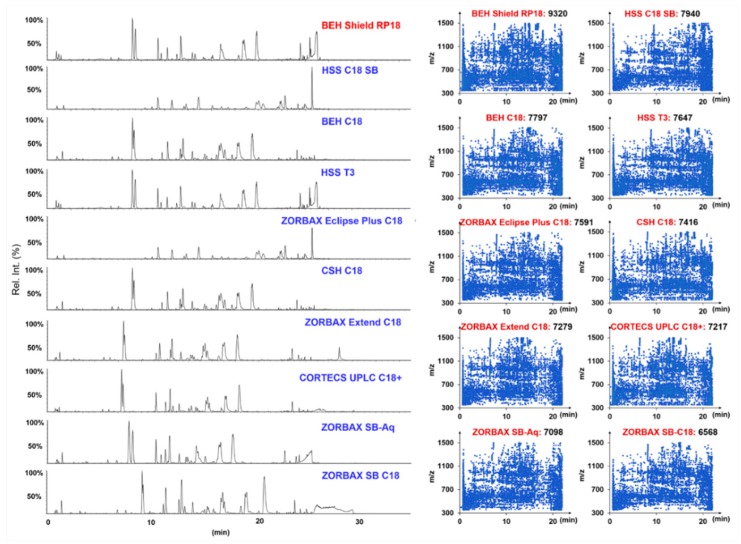
Comparison of ten different stationary phases (maintained at 35 °C) for the reversed-phase UHPLC separation of multicomponents from *Panax japonicus*: The left shows the base peak intensity (BPI) chromatograms obtained using ten candidate sub-2-µm particle-packed columns, and the right is the scatter plot of the components resolved by each chromatographic column.

The effects of different stationary phases on the separation of saponins from *P. japonicus* were further evaluated by considering the number of resolvable peaks and peak symmetry [17,31]. Ten UHPLC columns packed with sub-2-µm particles from two major vendors (Waters Corporation and Agilent Technologies), including HSS T3, HSS C18 SB, CSH C18, BEH Shield RP18, BEH C18, CORTECS UPLC C18+ (from Waters), ZORBAX Extend C18, ZORBAX SB-C18, ZORBAX Eclipse Plus C18, and ZORBAX SB-Aq (from Agilent), which we have utilized in the analysis of the flower buds of three *Panax* species [17], were examined at 35 °C (Figure 2). In structure, these different stationary phases are differentiated in the silica gel core (fully porous or core-shell), the bonding technologies, and the bonding groups. By eluting using the same gradient program, the BEH Shield RP18 (9320), HSS C18 SB (7940), BEH C18 (7797), HSS T3 (7647), and ZORBAX Eclipse Plus C18 (7591) columns were able to resolve more peaks than the other five. Impressively, the BEH Shield RP18 showed the best selectivity and high column efficiency on the separation of *P. japonicus* multicomponents. In addition, it exhibited almost the best separating capacity to Rg1/Re (comparable to the ZORBAX SB-Aq and HSS T3), a pair of ginsenosides diagnostic for discriminating different *Panax* species [29]. Accordingly, we selected the BEH Shield RP18 column in this experiment, consistent with the column we used in a previous chemical analysis of flower buds of *P. ginseng*, *P. quinquefolius*, and *P. notoginseng* [17] and the florets of *Carthamus tinctorius* [5]. In the next step, the effects of the column temperature varying from 25 °C to 40 °C were assessed by observing the overall resolution for ginsenosides (Figure S2). It was evident that increasing of the temperature could weaken the retention of *P. japonicus* saponins on the BEH Shield RP18 column and that the resolution of Rg1/Re became smaller. However, the influence in general was not significant. In case of the peaks that could be resolved, the temperature at 30 °C (8003) was better than the other settings (7793 peaks at 25 °C, 7197 at 35 °C, and 6809 at 40 °C). We thereby set the column temperature at 30 °C. Ultimately, the gradient elution program was slightly adjusted to obtain the optimal chromatography.

### 2.2. Optimization of the Parameters on Vion IM-QTOF Mass Spectrometer

Key parameters of the Vion IM-QTOF instrument (Waters Corporation), which may influence the ion response and the fragmentation, were further optimized. For the ion source, two parameters affecting the ion response, including spray voltage and cone voltage, had been optimized in our previous report [17], which were set at 1.0 kV and 20 V, respectively.

Collision energy is an important parameter that largely affects the quality of the MS^2^ spectra and the number of components that can be characterized. State-of-the-art mass spectrometers mostly enable ramp collision energy or mixed collision energies to acquire high-quality MS^2^ spectra for the precursors with a wide mass span. The Vion IM-QTOF hybrid high-resolution mass spectrometer can enable ramp collision energy (RCE) in MS^E^/HDMS^E^ [17,18] and mass dependent ramp collision energy (MDRCE) in the DDA mode (MS-MS/MS). No reports are currently available on how to optimize the MDRCE in DDA. Here, we present a “three-step” approach. The intensity ratios of sapogenin ions to precursors for 13 ginsenosides glycosylated with one to five sugars (mono-glycosides: ginsenosides Rh2 and Rh1; di-glycosides: chikusetsusaponin IVa, 24(*R*)-pseudoginsenoside F11, and ginsenoside Rg1; tri-glycosides: ginsenoside Ro, notoginsenoside R1, malonyl-floralginsenoside Re1; tetra-glycosides: ginsenosides Rb1, -Rc, and malonylginsenoside Rb1; and penta-glycosides: ginsenoside Ra1 and notoginsenoside R4), were used as the index for evaluation. These ginsenoside compounds involve the PPD-, PPT-, OA-, OT-, and malonyl-substituted subtypes, which could cover the structural diversity of the saponins of the entire *Panax* genus. From our previous work, we find the number of sugars in ginsenosides is the most important factor associated with the dissociation degree when a single level of collision energy is set.

**Figure 3:**
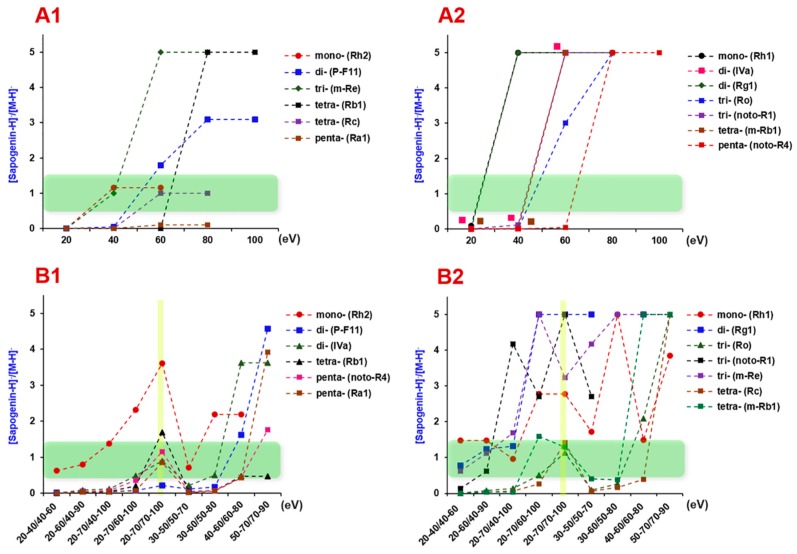
Optimization of the mass dependent ramp collision energy (MDRCE) for 13 ginsenosides that involve one to five sugars and represent five subclasses: (**A1**,**A2**) Determined at single levels of collision energy and (**B1**,**B2**) determined by setting different ranges of MDRCE values. For an easy depiction, the [Sapogenin–H]^−^ to [M–H]^−^ ratios higher than 5.0 were all recorded at 5.0. The best MDRCE value is marked in yellow in Figure 3B1,B2.

**Figure 4:**
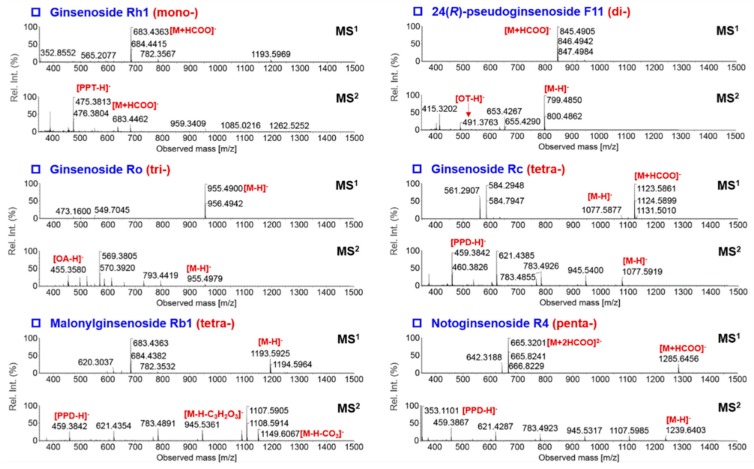
The MS^1^ and MS^2^ spectra of nine ginsenosides involving one to five sugars, showing the balanced MS/MS fragmentation by the optimized mass-dependent ramp collision energy.

In the first step, single levels of collision energy, varying from 20 eV to 140 eV (20 eV per step), were examined to probe into the appropriate collision energy suitable for the CID of ginsenosides with different numbers of sugars (one to five). We consider balanced MS^2^ spectra that contain both the precursors and sapogenin ions that can represent good-quality MS^2^ spectra beneficial to the purpose of structural elucidation. As evidenced from Figure 3A (the [Sapogenin–H]^–^/[M–H]^–^ ratio between 0.5–1.5 can partially demonstrate a balanced MS^2^ spectrum; the intensity ratio values were not given when CE > 100 eV as the precursor ions had been completely fragmented), we could draw a conclusion that ginsenosides glycosylated with more sugars were more difficult to be dissociated into the sapogenins. The collision energy of 20–60 eV was appropriate for one to tri-glycosidic ginsenosides (molecular weight (M.W.) < 1000 Da), while a higher collision energy of 40–80 eV was necessary for well fragmentation of ginsenosides with molecular weight >1000 Da (tetra- and penta-glycosides). Accordingly, in the second step, we set four groups of MDRCE settings with linearly increasing RCE for both low mass and high mass, involving 20–40 eV/40–60 eV, 30–50 eV/50–70 eV, 40–60 eV/60–80 eV, and 50–70 eV/70–90 eV (Figure 3B). Consequently, the MDRCE of 30–50 eV/50–70 eV was suitable for the CID of ginsenosides with 1–3 sugars, while the last two groups of MDRCE (40–60 eV/60–80 eV and 50–70 eV/70–90 eV) more suited for the dissociation of tetra- and penta-glycosidic ginsenosides. We could further alter the range of RCE for low mass and high mass, respectively, to enable better dissociation of mono- to penta-glycosidic ginsenosides. In the last step, another five groups of MDRCE were examined (20–60 eV/40–90 eV, 30–60 eV/ 50–80 eV, 20–70 eV/ 40–100 eV, 20–70 eV/60–100 eV, and 20–70 eV/70–100 eV). By considering the MS^2^ spectra quality of all 13 index ginsenosides, the MDRCE of 20–70 eV/70–100 eV was regarded as the best, under which the DDA-MS^2^ spectra of nine ginsenosides are exhibited in Figure 4.

On the other hand, RCE in HDMS^E^ was optimized as well. After comparing 6 different settings (30–90 eV, 30–100 eV, 30–120 eV, 40–100 eV, 50–100 eV, and 60–100 eV), we finally selected the RCE of 40–100 eV, under which good fragmentation for mono- to penta-glycosidic ginsenosides was accomplished.

### 2.3. Streamlined Workflows by Utilizing UNIFI to Automatedly Annotate both the DDA and HDMS^E^ Data

The versatile data processing platform UNIFI was applied to process and annotate the high-accuracy DDA and HDMS^E^ data to achieve the efficient and intelligent multicomponent characterization of *P. japonicus*. Streamlined workflows under the “Screening” analysis type, that is, “Accurate Mass Screening on DDA data” for DDA and “Accurate Mass Screening on IMS data” for HDMS^E^, facilitated by UNIFI (searching the in-house ginsenoside database containing 504 compounds) were employed. First, the DDA data were processed by the established data processing method, which generated a list of the characterized components as “Identified Components”, including various information such as observed *m*/*z*, formula, observed t_R_, mass error, adducts, etc. Second, various approaches (e.g., adduct filtering for carboxyl-free neutral ginsenosides, extracted ion chromatogram analysis based on the full-scan spectra for removing false positives that result from adducts or in-source fragmentation ions, MS/MS data analysis) were utilized to confirm the identification results and discriminating false positives. Third, the compounds listed in “Unknown Components” with response >10000 were analyzed manually based on the high-accuracy MS^1^ and MS^2^ data to enrich possible identifications with the mass not included in the in-house database. Fourth, the HDMS^E^ data were further processed using the corresponding method generating another table of identified components. The obtained table was further analyzed targeting two aims: (1) to add the CCS information to the compounds identified by DDA and (2) to enrich the identifications that were not achieved in DDA analysis. By these efforts, the coverage could be expanded due to the “Complementarity” of DDA and HDMS^E^ data to enable the comprehensive multicomponent characterization of TCM.

### 2.4. Complementarity of DDA and HDMS^E^ for the Comprehensive Multicomponent Characterization 

Aside from the complication of chemical matrix in an herb extract, diversity in the composition of ginsenoside precursors generated in the negative ESI mode otherwise affected the coverage of target components by DDA. Other forms of precursor ions, such as the adducts, dimers, and even various doubly charged ones, could trigger intensity-driven DDA, thus resulting in useless or repeating acquisition of the MS^2^ information. Using a major compound of *P. japonicus*, ginsenoside Ro (C_48_H_76_O_19_; theoretical deprotonated precursor calculated at *m*/*z* 955.4909) as a case, its full-scan spectrum displayed complex compositions of precursor ions: (1) deprotonated precursors at *m*/*z* 955.4907 ([M–H]^−^) as the base peak (100% intensity); (2) doubly charged FA-adducts ([M–2H+HCOOH]^2−^) at *m*/*z* 500.2449 (< 1% intensity); and (3) doubly charged trimers ([3M–2H]^2−^) at *m*/*z* 1433.7313 (10% intensity). These doubly charged precursors were also selected for MS/MS fragmentation in DDA, thus affecting the coverage (Figure S3). We should highlight that only a small proportion of the compounds in the “Unknown Components” list, obtained by analyzing the DDA data using UNIFI, corresponded to the target ginsenosides. In detail, among the 1212 components listed, 223 ones showed intensity higher than the threshold. Only 23 thereof, accounting for 1.90% of the total, could be tentatively characterized as ginsenosides (Table S2). The remaining can be classified into three cases: (i) mass error higher than the predefined tolerance (10 ppm); (ii) no neutral loss (NL) of sugars that are characteristic for the negative CID of ginsenosides [4,14,17,22,29,30]; and (iii) repeated acquisition of the isotope peaks of those identified components (Figure S4).

Considering that the MS^2^ spectra obtained by DDA can suffer from much less interference (DDA: the precursor ions fitting for the criteria are selected by quadrupole one by one for MS/MS fragmentation; MS^E^: full-range precursors are selected for MS/MS fragmentation), in the current work, the DDA data were firstly processed by UNIFI for structural elucidation and the HDMS^E^ data were comparatively analyzed to enrich new identifications. HDMS^E^ data can also offer the CCS values for most of the components identified by DDA. As a result, a total of 178 components were characterized from *P. japonicus*. Amongst them, 168 compounds were characterized by analyzing the DDA data (145 confirmed from the “Identified Components” and 23 from “Unknown Components”) and the other ten were newly added after analyzing the HDMS^E^ data. Therefore, these two data acquisition approaches exerted complementarity in the comprehensive multicomponent characterization of TCM. We had demonstrated the complementary role of Fast DDA and MS^E^ in characterizing quinochalcone C-glycosides from Carthamus tinctorius in one of our previous work [32].

Repeatability of both the DDA and HDMS^E^ approaches was evaluated by determining six copies of *P. japonicus* samples using eight index ginsenosides (20-O-Glc-Rf, noto-R1, Rg1, Re, noto-R4, Rb1, Rd, and chikusetsusaponin IVa). The repeatability of DDA and HDMS^E^ varied 0.77–3.63% and 1.56–4.67%, respectively. The “Identified Components” through six *P. japonicus* samples by processing the DDA and HDMS^E^ data using the streamlined workflows showed relative standard deviation (RSD) of 3.89% and 0.61%, respectively. All these data could indicate good repeatability for the established strategy applicable to the comprehensive multicomponent characterization of TCM.

### 2.5. Comprehensive Characterization of Multicomponents from P. japonicus by Analyzing both the DDA and HDMS^E^ Data

**Figure 5:**
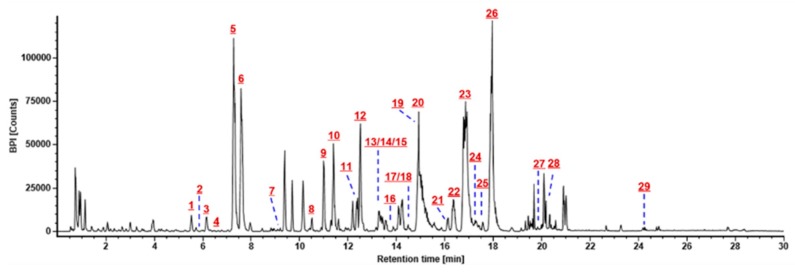
Base peak intensity (BPI) chromatogram of *Panax japonicus:* Peaks identified with the aid of reference compounds were marked **1**: 20-O-Glc-Rf; **2**: noto-Fp1; **3**: noto-R1; **4**: Re2; **5**: Rg1; **6**: Re; **7**: m-floral-Re1; **8**: p-F11; **9**: noto-R4; **10**: Rf; **11**: noto-R2 or F3; **12**: Rb1; **13**: Rg2; **14**: Rh1; **15**: m-Rb1; **16**: 20(R)-Rh1; **17**: Rb2; **18**: Rb3; **19**: F1; **20**: Ro; **21**: Rd; **22**: p-Rt1; **23**: chikusetsusaponin IV; **24**: m-Rd; **25**: noto-K or gypenoside XVII; **26**: chikusetsusaponin IVa; **27**: 20(S)- or 20(R)-Rg3; **28**: Rg5; and **29**: oleanolic acid.

By integrated analyses of both the DDA and HDMS^E^ data acquired in the negative ESI mode, we could identify or tentatively characterize 178 saponins from *P. japonicus* (Table S2), of which 29 were identified by comparison with the reference compounds (Figure 5). After further searching against the in-house database (consisting of 504 ginsenosides), 75 of these characterized ginsenosides may have not been isolated from the whole *Panax* genus. According to the difference of the sapogenin and the presence of malonyl substitution, these ginsenosides, characterized from *P. japonicus*, were classified into the PPD-, PPT-, OA-, OT-, and malonylated subtypes and the others. 

#### 2.5.1. Characterization of OA-Ginsenosides Of the *Panax* Species

**Figure 6:**
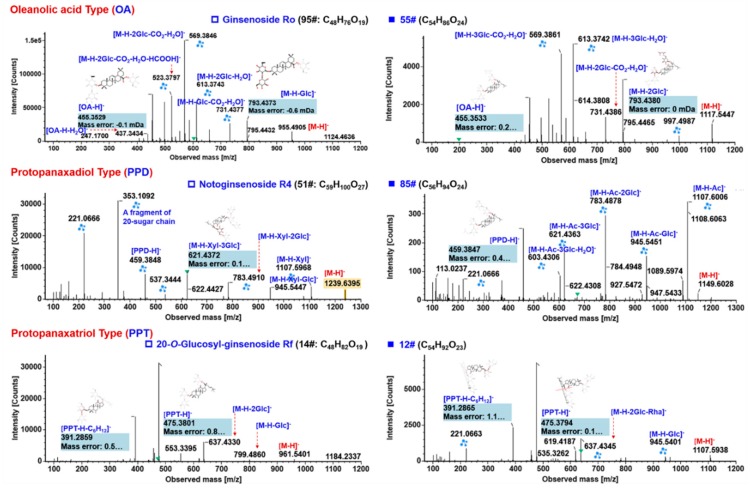
Annotation of the MS^2^ spectra of oleanolic acid (OA)-, protopanaxadiol (PPD)-, and protopanaxatriol (PPT)-ginsenosides (the left column) from *P. japonics* by UNIFI^TM^, showing the characteristic fragmentations and their application to characterizing unknown components (the right column).

*P. japonicus* is characteristic for the high content of OA-saponins (such as ginsenoside Ro/chikusetsusaponin V, pseudoginsenoside Rt1, chikusetsusaponins IV, and IVa) [24,26]. In this work, we could characterize 76 OA-ginsenosides (42.7% of the total amount), of which **95**# (Ro), **122**# (p-Rt1), **127**# (IV), and **135**# (IVa) were identified with the aid of reference compounds. One identification point for OA-ginsenosides was the insular deprotonated precursor (without remarkable FA-adducts). Firstly, the negative CID-MS^2^ features of the reference compound ginsenoside Ro (consistent with **95**# in Table S2: t_R_, 14.83 min) was interpreted (Figure 6). In structure, Ro is featured by 3-GlurA as the inner sugar and 28-Glc glycosylated at COOH. Its CID exhibited structure-related fragmentations. Aside from the easy elimination of 28-Glc (*m*/*z* 793.4373) and the external Glc + H_2_O of 3-sugar chain (*m*/*z* 613.3743), the top three most intense MS^2^ fragments were observed at *m*/*z* 569.3846, 455.3529, and 523.3797, of which *m*/*z* 455.3529 was the deprotonated OA (another fragment of OA was detected at *m*/*z* 437.3434 due to the neutral loss of H_2_O). The other two ions, *m*/*z* 569 ([M–H–2Glc–CO_2_–H_2_O]^−^) and 523 ([M–H–2Glc–CO_2_–H_2_O–HCOOH]^−^), were the results of crossing cleavages on 3-GlurA after the elimination of two Glc, which could be diagnostic for the rapid characterization of an OA-3-GlurA substructure. Clear assignment of the fragmentation ions of ginsenoside Ro was beneficial to elucidating the structures of the other unknown OA-saponins, such as compound **55**# (t_R_, 11.62 min; C_54_H_86_O_24_) which gave abundant precursor ion at *m*/*z* 1117.5458. By comparing its MS^2^ spectrum with that of Ro, similar fragments (*m*/*z* 955, 793, 731, 613, 569, 523, 455, and 437) were detected, which could suggest the presence of a structure similar to Ro. The transition from the precursor to the ion *m*/*z* 955.4945 indicated an additional Glc residue. We have searched this structure, OA-GlurA-Glc-Glc-Glc, in our in-house database, and surprisingly, no hit was found. More importantly, by searching the molecular formula in SciFinder, we can confirm it is a new ginsenoside compound. Notably, despite the saponins having a ursolic acid (UA) sapogenin (an isomer of OA) that was isolated from this medicinal herb [25], it was impossible to differentiate between UA and OA under the current condition only with the MS data. Therefore, OA was used in the identifications to represent the sapogenins observed at *m*/*z* 455.35 (Table S2).

#### 2.5.2. Characterization of PPD-/PPT-Ginsenosides

Ginsenosides with a PPD or PPT sapogenin represent the most common saponins for various *Panax* species [19]. We could characterize 15 PPD-type (8.4% of the total amount) and 33 PPT-type (18.5%) ginsenosides from *P. japonicus.* Neutral PPD- and PPT-ginsenosides could readily form FA-adducts as the predominant precursors, which is rather different from the OA-saponins. Concomitant FA-adduct and the deprotonated precursor, together with the typical sapogenin ions at *m*/*z* 459.38 and 475.38, can assist the rapid characterization of ginsenosides of these two subtypes. Consistent with our previous researches [4,14,17,22,29,30], the negative CID of ginsenoside precursors could readily eliminate the attached sugars and generate the product ions of sapogenins or their related secondary species. However, the obtained MS^2^ fragments are not as diagnostic for identifying the distribution and for assigning the glycosylation sites as the positive-mode product ions (Z_0α_^+^ and C_nβ_^+^) [4,33]. Notoginsenoside R4 (corresponding to **51**#: t_R_, 10.97 min; C_59_H_100_O_27_) is a PPD-type ginsenoside having five sugars attached to 3-OH and 20-OH, respectively. Due to the use of MDRCE, much more balanced MS^2^ spectra, showing various product ions involving the precursor ion, sugar-elimination resulting fragments, sapogenin ion, and even the sugar chain fragments, were obtained (Figure 6). CID-MS/MS of the precursor at *m*/*z* 1239.6430 generated rich and diversified product ions as a result of sugar eliminations. The ions of *m*/*z* 1107.5968, 945.5447, 783.4910, 621.4372, and 459.3848 were consistent with the subsequential cleavages of Xyl (132.04 Da) and four Glc (162.05 Da). In addition, the base peak ion at *m*/*z* 353.1092 as well as the second most intense ion *m*/*z* 221.0666 were the fragments of the 20-GlcGlcXyl or 3-GlcGlc sugar chains. In the case of an unknown PPD-type saponin **85**# (t_R_, 13.87 min; C_56_H_94_O_24_), very similar MS^2^ fragments to those of noto-R4 were observed (*m*/*z* 1107.6006, 945.5451, 783.4878, 621.4363, and 459.3847). Another typical product ion at *m*/*z* 375.2909 was the secondary fragment of the PPD sapogenin by eliminating C_6_H_12_ on the C17-side chain [4,22]. It indicated that this compound contained the same PPD skeleton and four Glc as Rb1 (**65**#: t_R_, 12.49 min; C_54_H_92_O_23_) and that the different part was an acetyl substituent (+42.0106 Da) which was easily eliminated by CID-MS^2^. These structural features of compound **85**# matched 6”-O-acetylginsenoside Rb1, an immunosuppressive saponin isolated from *P. quinquefolius* [34], in the in-house ginsenoside database. Its stronger retention than Rb1 on the RP column could also testify to its identification because of the presence of acetylation. On the other hand, the CID-MS^2^ behaviors of PPT-ginsenosides were extremely analogous to those of the PPD-type. The largest difference was the generation of the sapogenin ion at *m*/*z* 475.38 and its secondary fragment *m*/*z* 391.29 (by eliminating C_6_H_12_). The MS^2^ spectra of the reference compound 20-O-Glc-Rf (corresponding to **14**#; t_R_, 5.53 min) could provide evidence to reflect the fragmentation features of PPT-type ginsenosides (Figure 6). For another unknown compound **12**# (t_R_, 4.85 min; C_54_H_92_O_23_), it was primarily characterized as an isomer of ginsenoside Rb1. However, its MS^2^ spectrum suggested a PPT-type saponin as rich fragments at *m*/*z* 475.3794 and 391.2865 were observed. The transitions 1107 > 945, 945 > 799, 799 > 637, and 637 > 475 indicated the presence of three Glc and one Rha. By searching against the in-house database, two hits of yesanchinoside E and ginsenoside Re8 were found. The other PPD- and PPT-ginsenosides were characterized in the same manner, with their information given in Table S2.

#### 2.5.3. Characterization of OT-Ginsenosides

**Figure 7:**
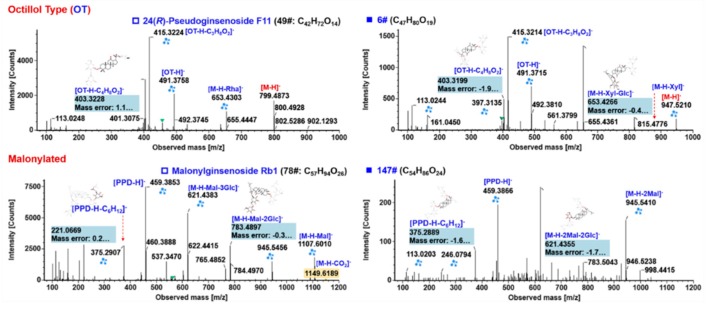
Annotation of the MS^2^ spectra of octillol (OT)- and malonylated ginsenosides (the left column) from *P. japonics* by UNIFI^TM^, showing the characteristic fragmentations and their application to characterizing unknown components (the right column).

Ginsenosides belonging to the OT type bear a characteristic sapogenin (C_30_H_52_O_6_) with a tetrahydrofuran ring, which are widely distributed in the *Panax* genus such as *P. quinquefolius* and *P. japonicus* [35]. A total of 11 OT-ginsenosides (6.2% of the total amount) were characterized. 24(R)-Pseudoginsenoside F11 (corresponding to compound **49**#: t_R_, 10.53 min; C_42_H_72_O_14_) is a marker compound to differentiate *P. quinquefolius* from *P. ginseng* [29]. Its MS^2^ spectrum exhibited rich fragments due to the successive neutral loss of Rha (*m*/*z* 653.4303) and Glc (*m*/*z* 491.3758). The product ion of *m*/*z* 491.38 ([OT–H]^−^) as well as its secondary fragments *m*/*z* 415.32 ([OT–H–C_3_H_8_O_2_]^−^) and 403.32 ([OT–H–C_4_H_8_O_2_]^−^), due to the fragmentations on the tetrahydrofuran ring, were the most important diagnostic ions for identifying an OT-ginsenoside (Figure 7). In the case of compound **6**# (t_R,_ 2.98 min; C_47_H_80_O_19_) which generated an abundant FA-adduct precursor ion at *m*/*z* 993.5266, almost the same fragments at *m*/*z* 653, 491, 415, and 403 as those of 24(R)-p-F11 were observed. The other transitions, 947 > 815 and 815 > 653, could suggest the presence of pentose (Xyl was used to express all the pentoses consistent with the neutral loss of 132.04 Da) and Glc. Therefore, it could be characterized as OT-Glc-Rha-Xyl. Only one hit, vinaginsenoside R6 [36], was found by searching our ginsenoside database.

#### 2.5.4. Characterization of Malonylated Ginsenosides

Ginsenosides from the *Panax* genus experience various modifications, such as malonylation which can increase the polarity of ginsenosides [19]. Malonylginsenosides have shown potential anti-diabetic activity in vitro [37,38]. In this work, 12 malonylginsenosides (6.7% of the total amount) were characterized. Due to the vulnerability of the ester bond, malonylginsenosides were featured by the easy neutral loss of CO_2_ and C_3_H_2_O_3_ (representing the whole malonyl substituent), which even readily occurred in full-scan MS^1^ spectra as in-source fragmentation. Special attention should be paid to the determination of the precursor ions for malonylginsenosides, as they may be incorrectly identified as acetylginsenosides as the deprotonated molecules easily eliminated CO_2_ in full-scan MS^1^. We have reported two potent strategies for the in-depth characterization of malonylginsenosides from Ginseng extracts [30,39]. In general, the malonyl group could be readily eliminated from the deprotonated molecule, while the CID of the remaining neutral ginsenosides was the same as those PPD-/PPT-ginsenosides. These CID features can be evidenced from the MS/MS fragmentation of the reference compound, m-Rb1 (consistent with compound **78**#: t_R_, 13.47 min; C_57_H_94_O_26_; Figure 7). The product ions of the neutral part (*m*/*z* 1107. 6010, 945.5456, 783.4897, 621.4383, 537.3470, 458.3853, 375.2907, 323.0984, and 221.0669) were almost identical to those generated by CID-MS/MS of Rb1. To an unknown compound **147**# (t_R_, 19.24 min; C_54_H_86_O_24_), the remaining neutral part after neutral loss of 172 Da, corresponding to two malonyl groups, gave the MS^2^ product ions as those of ginsenoside Rd (*m*/*z* 945.5410, 927.5246, 783.5043, 621.4355, 459.3866, and 375.2889). Accordingly, we could primarily characterize it as dimalonyl-ginsenoside Rd. Only one hit, malonyl-floralginsenoside Rd6 [37], the unique dimalonyl-ginsenoside we had isolated from the flower bud of *P. ginseng*, was matched in the in-house ginsenoside database.

Additionally, in total, 31 ginsenosides (17.4% of the total amount) were identified or tentatively characterized with diverse sapogenins. Their sapogenins vary among hydroxylated PPT (PPT + O), H_2_O-addition PPT (PPT + H_2_O), hydroxylated and H_2_O-addition PPT (PPT + O + H_2_O), 7-hydroxy-5-ene-PPD, hydroxylated OA (OA + O), OA-methyl ester (OA + CH_2_), DD II, and dehydrated PPT. These saponins with unusual sapogenins may be the source for discovering novel ginsenoside molecules [19]; however, their structures are difficult to be fully established only by the MS data, which deserve further investigations in our future work.

**Figure 8:**
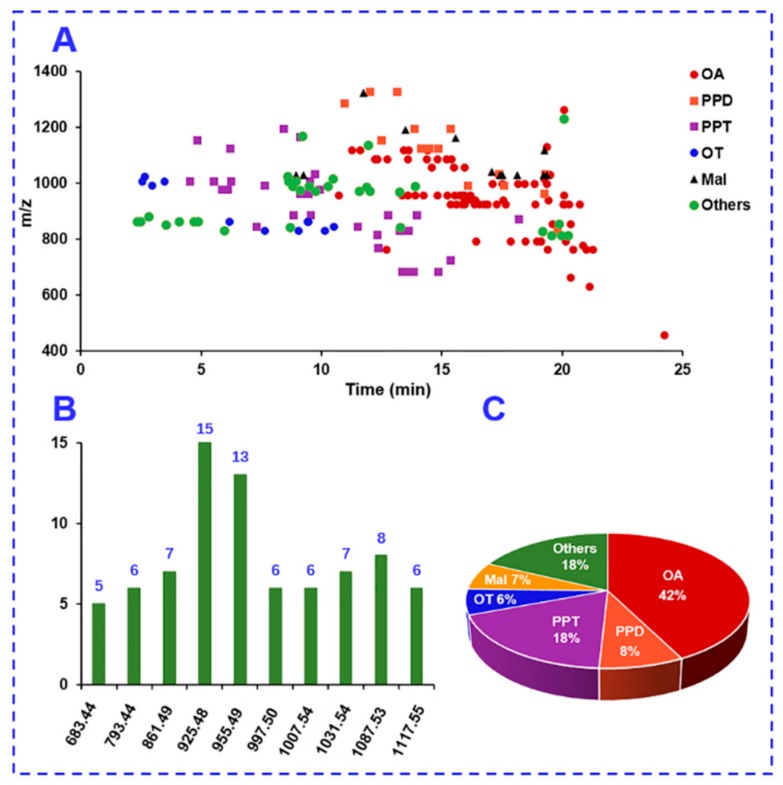
A summary of the components characterized from *Panax japonicus* in this work: (**A**) A 2-D scatter plot with *m*/*z* VS t_R_ (min); (**B**) popular isomerism showing the first 10 *m*/*z* values with the highest number of isomers; and (**C**) a pie chart displaying the proportion of each subtype of ginsenosides.

The structure features of the saponins identified in *P. japonicus* are outlined. Figure 8A shows a scatter plot of these 178 saponins with *m*/*z* VS t_R_. Isomerism is very popular for *P. japonicus* saponins [19,22], with top 10 masses with the highest number of isomers as exhibited in Figure 8B (not less than five isomers for each mass). Impressively, the amounts of isomers for *m*/*z* 925.48 and 955.49 both exceeded ten. The proportion of different subtypes of ginsenosides characterized from *P. japonicus* is shown in a pie chart in Figure 8C. Notably, isomerism is very popular for ginsenosides identified from various *Panax* species. The exact assignment of the known ginsenoside isomers in ginsenoside analyses renders a great challenge. In addition to the MS data, more dimensions of information should be determined to favor the discrimination of isomeric ginsenosides, such as the predicted t_R_ or CCS, which will be involved in the future work.

## 3. Materials and Methods

### 3.1. Reagents and Chemicals

In total, 60 compounds (Figure 1 and Table S1), involving 20(*S*)-protopanaxatriol (**1**), ginsenosides F1 (**2**), -Rh1 (**3**), 20(*R*)-ginsenoside Rh1 (**4**), 6-*O*-*β*-D-(6’-acetyl)-glucopyranosyl-24-en-dammar-3,6,12,20(*S*)-tetraol (**5**), ginsenosides Rg1 (**6**), -F3 (**7**), 20(*S*)-sanchirhinoside A3 (**8**), ginsenoside F5 (**9**), 6-*O*-(*β*-D-glucopyranosyl)-20-*O*-(*β*-D-xylopyranosyl)-3,6α,12,20(*S*)-tetrahydroxydammar-24-ene (**10**), pseudoginsenoside Rt3 (**11**), notoginsenoside R2 (**12**), 20(*R*)-notoginsenoside R2 (**13**), ginsenoside Rg2 (**14**), notoginsenoside Rt (**15**), ginsenoside Rf (**16**), notoginsenoside R1 (**17**), ginsenoside Re (**18**), vinaginsenoside R4 (**19**), 20-*O*-glucosyl-ginsenoside Rf (**20**), notoginsenosides N (**21**), -Fp1 (**22**), ginsenosides Re2 (**23**), -Re3 (**24**), malonyl-floralginsenoside Re1 (**25**), ginsenoside Rh2 (**26**), 20(*R*)-ginsenoside Rh2 (**27**), compound K (**28**), ginsenosides F2 (**29**), -Rg3 (**30**), 20(*R*)-ginsenoside Rg3 (**31**), notoginsenoside K (**32**), ginsenoside Rd (**33**), gypenoside XVII (**34**), malonyl-floralginsenoside Rd5 (**35**), malonyl-ginsenoside Rd (**36**), ginsenosides Rb2 (**37**), -Rb3 (**38**), -Rc (**39**), -Rb1 (**40**), malonyl-ginsenosides Rc (**41**), -Rb2 (**42**), -Rb1 (**43**), notoginsenoside R4 (**44**), ginsenosides Ra1 (**45**), -Ra2 (**46**), notoginsenoside T (**47**), oleanolic acid (**48**), chikusetsusaponin IVa (**49**), ginsenoside Ro (**50**), chikusetsusaponin IV (**51**), pseudoginsenoside Rt1 (**52**), 24(*R*)-pseudoginsenosides Rt5 (**53**), -F11 (**54**), ginsenosides Rk1 (**55**), -Rg5 (**56**), 5,6-didehydroginsenoside Rb1 (**57**), ginsenosides Rk3 (**58**), -Rh4 (**59**), and notoginsenoside T5 (**60**), purchased from Shanghai Standard Biotech. Co., Ltd. (Shanghai, China) or isolated from the root of *P. ginseng* (the structures were established by HRMS and NMR; HPLC-UV was used for purity determination) were used as the reference compounds in this work. Acetonitrile, methanol (Fisher, Fair lawn, NJ, USA), formic acid (FA), and ammonium acetate (AA; Sigma-Aldrich, St. Louis, MO, USA) were LC-MS grade. Ultra-pure water was in-house prepared using a Milli-Q water purification system (Millipore, Bedford, MA, USA). The sample of *Panax japonicus* C. A. Meyer analyzed in this work was purchased from Sichuan Province of China. The voucher specimen of *P. japonicus* (PJ-20180910) was deposited in the authors’ lab in Tianjin University of Traditional Chinese Medicine (Tianjin, China).

### 3.2. Sample Preparation

An easy-to-implement ultrasonic extraction method was utilized. In detail, 25 mg of the accurately weighed powder of *P. japonicus* was soaked in 5 mL of 70% aqueous methanol (*v*/*v*), which was further extracted in a water bath at 25 °C with ultrasound assistance for 1 h. The lost weight was compensated with 70% methanol. After 10 min of centrifugation at a rotation speed of 14,000 rpm, the supernatant was used as the test solution, with the final concentration at 5 mg/mL (the same concentration we previously applied in Reference [17]).

### 3.3. UHPLC/IM-QTOF-MS

Multicomponent profiling and characterization of *P. japonicus* were performed on an ACQUITY UPLC I-Class/Vion IM-QTOF system (Waters, Milford, MA, USA). A BEH Shield RP18 (2.1 × 100 mm, 1.7 μm) hyphenated with a VanGuard Pre-column (2.1 × 50 mm, 1.7 μm) maintained at 30 °C was used for the chromatographic separation. A binary mobile phase, composed by CH_3_CN (organic phase: A) and 0.1% formic acid in H_2_O (water phase: B), was employed at a flow rate of 0.3 mL/min in consistency with an optimal gradient program: 0–2 min, 15–20% (A); 2–7 min, 20–23% (A); 7–8 min, 23–29% (A); 8–13 min, 29–32% (A); 13–18 min, 32–35% (A); 18–19 min, 35–70% (A); 19–23 min, 70–84% (A); 23–23.5 min, 84–95% (A); and 23.5–26.5 min, 95% (A). A 5-min re-equilibration time was set between the successive injections. Three microliters of the test solution was injected for UHPLC separation. A “purge-wash-purge” cycle was set on the autosampler, using 10% CH_3_CN in H_2_O (*v*/*v*) as the purge solvent and 50% CH_3_CN in H_2_O as the wash solvent, to minimize the carry-over between injections. 

High-accuracy MS data were acquired on a Vion IM-QTOF mass spectrometer in both the negative DDA (MS-MS/MS) and HDMS^E^ mode (Waters). The LockSpray ion source was equipped using the following parameters: capillary voltage, −1.0 kV; cone voltage, 20 V; source offset, 80 V; source temperature, 120 °C; desolvation gas temperature, 500 °C; desolvation gas flow (N_2_), 800 L/h; and cone gas flow (N_2_), 50 L/h. Default parameters were defined for the travelling wave IM separation. The QTOF analyzer scanned over a mass range of *m*/*z* 350−1500 at a low collision energy of 6 eV for both HDMS^E^ and DDA at 0.3 s per scan (MS^1^). The ramp collision energy (RCE) of 40–100 eV was set in HDMS^E^. When TIC (total ion chromatogram) intensity exceeded 5000 detector counts, MS/MS fragmentation of the five most intense precursors was automatedly triggered in DDA mode under mass-dependent ramp collision energy (MDRCE) of 20–70 eV in low mass ramp settings and 70–100 eV in high mass ramp settings (scan range in MS^2^, *m*/*z* 100–1500). The MS/MS acquisition stopped if either TIC dropped below 100 detector counts or time exceeded 0.2 s. No dynamic peak exclusion was set for DDA. MS data calibration was conducted by constantly infusing the leucine enkephalin (LE; Sigma-Aldrich; 200 ng/mL) at a flow rate of 10 µL/min [17]. Calibration of CCS was conducted according to the manufacture’s guidelines using a mixture of calibrants [40].

### 3.4. Date Processing

The uncorrected DDA and HDMS^E^ data in Continuum were further processed using UNIFI 1.9.3.0 (Waters). The UNIFI software performed data correction, peak picking, and peak annotation efficiently. Key parameters set in UNIFI were as follows. Find 4D Peaks (only set in HDMS^E^): High-energy intensity threshold, 100.0 counts; low-energy intensity threshold, 5.0 counts. Target by mass: Target match tolerance: 10.0 ppm; screen on all isotopes in a candidate, generate predicted fragments from structure, and look for in-source fragments were enabled; fragment match tolerance, 10.0 ppm. Adducts: Negative adducts including +HCOO^−^, −H^−^. Lock Mass: Combine width, 3 scans; mass window, 0.5 *m*/*z*; reference mass, 554.2620; reference charge, –1.

## 4. Conclusions

To enable the comprehensive profiling and characterization of multicomponents from TCM, we attempted to integrate two different data acquisition approaches, DDA and HDMS^E^, to extend the coverage by means of the powerful platform, UHPLC/IM-QTOF-MS. Targeting a herbal medicine derived from the *Panax* genus, the UHPLC condition and the MS detection by Vion IM-QTOF high-resolution mass spectrometer were optimized. In particular, we presented a “three-step” approach to optimize the mass-dependent ramp collision energy (MDRCE) in DDA. As a result, good resolution of the saponins from *P. japonicus* was accomplished using a reversed-phase BEH Shield RP18 column eluted by CH_3_CN/0.1% FA-H_2_O. The negative-mode DDA and HDMS^E^ both could generate rich product ions useful for the structural elucidation and, more importantly, exerted complementarity. The potent data-processing platform UNIFI^TM^, by matching with an in-house ginsenoside database (recording 504 known compounds and 60 ginsenoside reference compounds), could efficiently annotate the high-accuracy DDA and HDMS^E^ data. By streamlined workflows, we could identify or tentatively characterize 178 saponins from *P. japonicus*, and 75 thereof may have not been isolated from the *Panax* genus, which can demonstrate the good performance by combining two data acquisition methods. The results obtained enable deep insight into the complex composition of the metabolites in this medicinal herb. The integration of DDA and MS^E^ renders a powerful strategy for the systematic multicomponent characterization for TCM.

## Supplementary Materials

The following are available online: Figure S1: Comparison of the influence of formic acid (0.1% FA; A) or ammonium acetate (3 mM AA; B) as the additive in the mobile phase on the resolution of multicomponents from an extract of *Panax japonicus*; Figure S2: Comparison of the influence of column temperature (25–40 °C) on the BEH Shield RP18 column for the resolution of ginsenosides from *Panax japonicus*; Figure S3: Illustration for the diversity of ginsenoside precursors in the negative ESI mode using ginsenoside Ro as a case, a major OA-type saponin in *Panax japonicus*; Figure S4: A typical case of repeating acquisition of the MS^2^ information for the isotope peak of the identified components by DDA listed as “Unknown Components”; Table S1: Information of the 60 ginsenoside reference compounds used in this work; Table S2: Information of the 178 saponins identified in *Panax japonicus* by integrated analyses of the DDA and HDMS^E^ data.
