# Supplementary material for: Integration of Data-Dependent Acquisition (DDA) and Data-Independent High-Definition MSE (HDMSE) for the Comprehensive Profiling and Characterization of Multicomponents from Panax japonicus by UHPLC/IM-QTOF-MS
Source: Molecules. 2019 Jul 25;24(15):2708. doi: 10.3390/molecules24152708 (PMC6695638; doi:10.3390/molecules24152708)
Supplement: Supplementary file 1 [file molecules-24-02708-s001.zip › Supplementary Materials.pdf]

<**Molecules**>

Electronic Supplementary Materials

Integration of data-dependent acquisition (DDA) and data-independent high-definition MS^E (HDMS^E) for the comprehensive profiling and characterization of multicomponents from *Panax japonicus* by UHPLC/IM-QTOF-MS

Chunxia Zhang [1,2,†], Tiantian Zuo [1,2,†], Xiaoyan Wang [1,2,†], Hongda Wang [1,2], Ying Hu [1,2], Zheng Li [1,3], Weiwei Li [1,2], Li Jia [1,2], Yuexin Qian [1,2], Wenzhi Yang [1,2,*] and Heshui Yu [1,3,*]

[1] Tianjin State Key Laboratory of Modern Chinese Medicine, Tianjin University of Traditional Chinese Medicine, 312 Anshanxi Road, Tianjin 300193, China

[2] Tianjin Key Laboratory of TCM Chemistry and Analysis, Tianjin University of Traditional Chinese Medicine, 312 Anshanxi Road, Tianjin 300193, China

[3] College of Pharmaceutical Engineering of Traditional Chinese Medicine, Tianjin University of Traditional Chinese Medicine, Tianjin 300193, China

[*] Correspondence: wzyang0504@tjutcm.edu.cn, Tel.: +86-022-5979-1833 (W.Y.); hs_yu08@163.com (H.Y.)

[†] These authors contributed equally to this work.

Contents

Figure S1 Comparison of the influence of formic acid (0.1% FA; **A**) or ammonium acetate (3 mM AA; **B**) as the additive in mobile phase on the resolution of multicomponents from an extract of *Panax japonicus*.

Figure S2 Comparison of the influence of column temperature (25–40 °C) on the BEH Shield RP18 column for the resolution of ginsenosides from *Panax japonicus*.

Figure S3 Illustration for the diversity of ginsenoside precursors in the negative ESI mode, using ginsenoside Ro as a case, a major OA-type saponin in *Panax japonicus*.

Figure S4 A typical case of repeating acquisition of the MS^2 information for the isotope peak of the identified components by DDA listed as "Unknown Components".

Table S1 Information of 60 ginsenoside reference compounds used in this work.

Table S2 Information of the 178 saponins identified in *Panax japonicus* by integrated analyses of the DDA and $HDMS^E$ data.

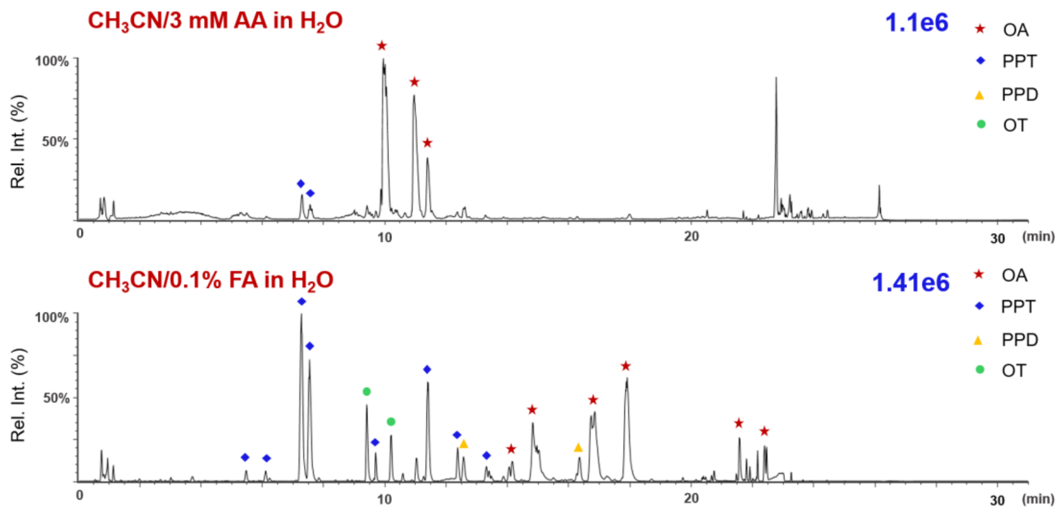

Figure S1 Comparison of the influence of formic acid (0.1% FA; **A**) or ammonium acetate (3 mM AA; **B**) as the additive in mobile phase on the resolution of multicomponents from an extract of *Panax japonicus*.

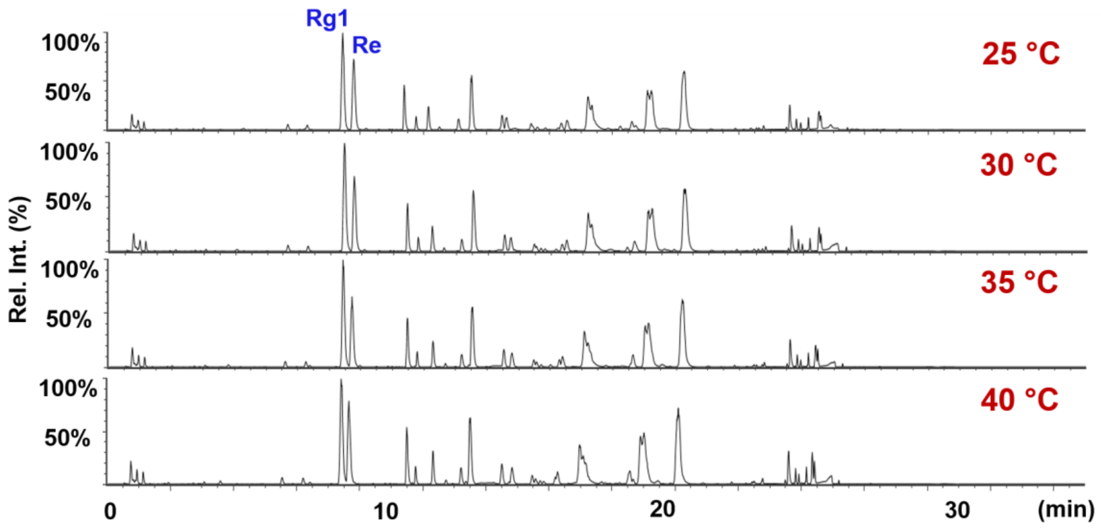

Figure S2 Comparison of the influence of column temperature (25–40 °C) on the BEH Shield RP18 column for the resolution of ginsenosides from *Panax japonicus*.

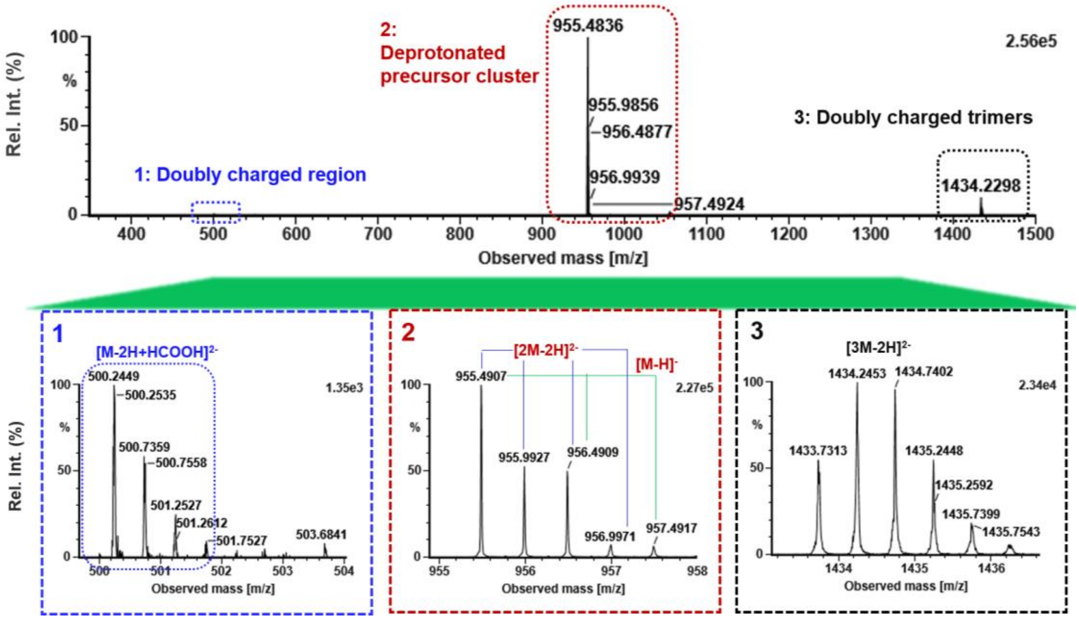

Figure S3 Illustration for the diversity of ginsenoside precursors in the negative ESI mode, using ginsenoside Ro as a case, a major OA-type saponin in *P. japonicus*.

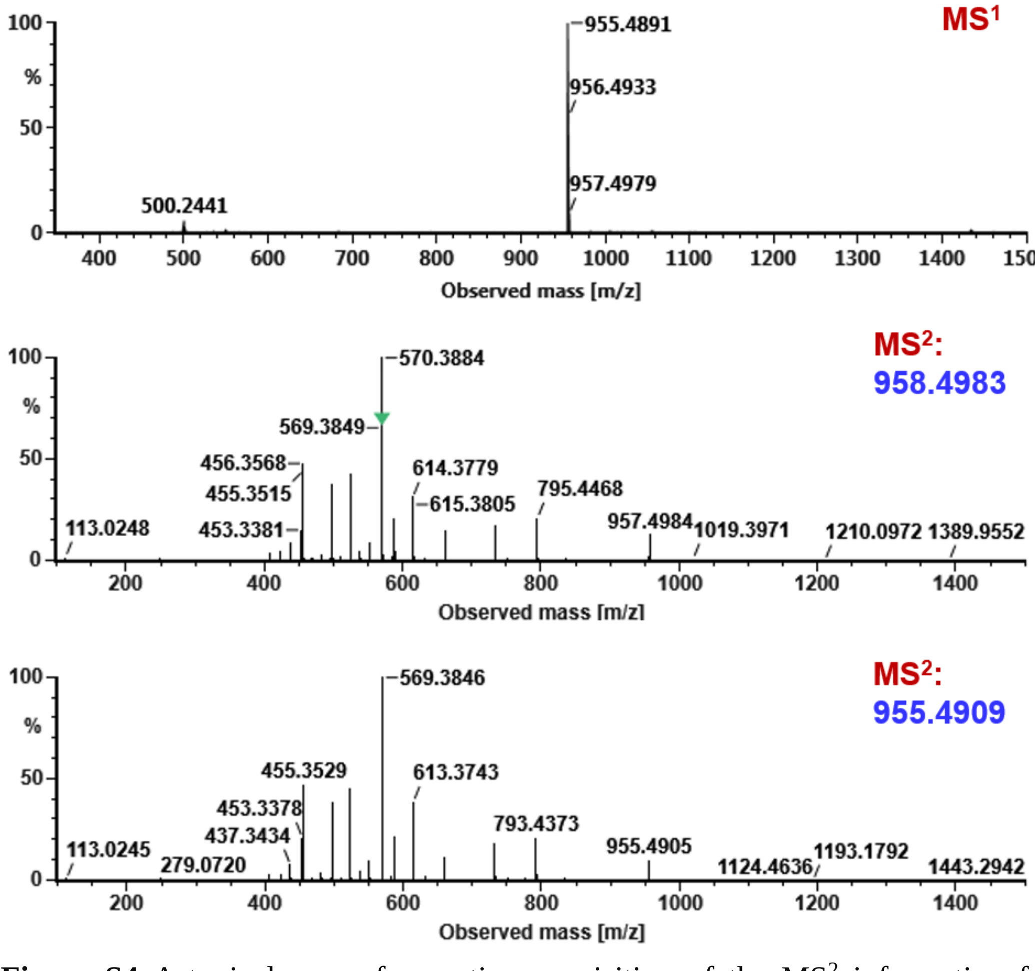

Figure S4 A typical case of repeating acquisition of the MS^2 information for the isotope peak of the identified components by DDA listed as “Unknown Components”.

Table S1 Information of 60 ginsenoside reference compounds used in this work.

No.	Compound	M.F.	Exact mass	Subclass
1	20(*S*)-protopanaxatriol	$C_{30}H_{52}O_{4}$	476.3866	
2	ginsenoside F1	$C_{36}H_{62}O_{9}$	638.4394	
3	ginsenoside Rh1	$C_{36}H_{62}O_{9}$	638.4394	
4	20(R)-ginsenoside Rh1	$C_{36}H_{62}O_{9}$	638.4394	
5	Compound I	$C_{38}H_{64}O_{10}$	680.4499	
6	ginsenoside Rg1	$C_{42}H_{72}O_{14}$	800.4922	
7	ginsenoside F3	$C_{41}H_{70}O_{13}$	770.4816	
8	sanchinoside A3	$C_{41}H_{70}O_{13}$	770.4816	
9	ginsenoside F5	$C_{41}H_{70}O_{13}$	770.4816	
10	compound II	$C_{41}H_{70}O_{13}$	770.4816	
11	pseudoginsenoside Rt3	$C_{41}H_{70}O_{13}$	770.4816	
12	notoginsenoside R2	$C_{41}H_{70}O_{13}$	770.4816	
13	20(*R*)-notoginsenoside R2	$C_{41}H_{70}O_{13}$	770.4816	PPT
14	ginsenoside Rg2	$C_{42}H_{72}O_{13}$	784.4973	
15	notoginsenoside Rt	$C_{44}H_{74}O_{15}$	842.5028	
16	ginsenoside Rf	$C_{42}H_{72}O_{14}$	800.4922	
17	notoginsenoside R1	$C_{47}H_{80}O_{18}$	932.5345	
18	ginsenoside Re	$C_{48}H_{82}O_{18}$	946.5501	
19	vinaginsenoside R4	$C_{48}H_{82}O_{18}$	962.5450	
20	20-*O*-glucosylginsenoside Rf	$C_{48}H_{82}O_{18}$	962.5450	
21	notoginsenoside N	$C_{48}H_{82}O_{18}$	962.5450	
22	notoginsenoside Fp1	$C_{47}H_{80}O_{18}$	932.5345	
23	ginsenoside Re2	$C_{48}H_{82}O_{18}$	962.5450	
24	ginsenoside Re3	$C_{48}H_{82}O_{18}$	962.5450	
25	malonyl-floralginsenoside Re1	$C_{51}H_{84}O_{21}$	1032.5505	
26	ginsenoside Rh2	$C_{36}H_{62}O_{8}$	622.4445	
27	20(*R*)-ginsenoside Rh2	$C_{36}H_{62}O_{8}$	622.4445	
28	compound K	$C_{36}H_{62}O_{8}$	622.4445	
29	ginsenoside F2	$C_{42}H_{72}O_{13}$	784.4973	
30	20(*S*)-ginsenoside Rg3	$C_{42}H_{72}O_{13}$	784.4973	
31	20(*R*)-ginsenoside Rg3	$C_{42}H_{72}O_{13}$	784.4973	
32	notoginsenoside K	$C_{48}H_{82}O_{18}$	946.5501	
33	ginsenoside Rd	$C_{48}H_{82}O_{18}$	946.5501	
34	gypenoside XVII	$C_{48}H_{82}O_{18}$	946.5501	PPD
35	malonyl-floralginsenoside Rd5	$C_{51}H_{84}O_{21}$	1032.5505	
36	malonyl-ginsenoside Rd	$C_{51}H_{84}O_{21}$	1032.5505	
37	ginsenoside Rb2	$C_{53}H_{90}O_{22}$	1078.5924	
38	ginsenoside Rb3	$C_{53}H_{90}O_{22}$	1078.5924	
39	ginsenoside Rc	$C_{53}H_{90}O_{22}$	1078.5924	
40	ginsenoside Rb1	$C_{54}H_{92}O_{23}$	1108.6029	
41	malonyl-ginsenoside Rc	$C_{56}H_{92}O_{25}$	1164.5928	

42	malonyl-ginsenoside Rb2	$C_{56}H_{92}O_{25}$	1164.5928	
43	malonyl-ginsenoside Rb1	$C_{57}H_{94}O_{26}$	1194.6033	
44	notoginsenoside R4	$C_{59}H_{100}O_{27}$	1240.6452	
45	ginsenoside Ra1	$C_{58}H_{98}O_{26}$	1210.6346	
46	ginsenoside Ra2	$C_{58}H_{98}O_{26}$	1210.6346	
47	notoginsenoside T	$C_{64}H_{108}O_{31}$	1372.6875	
48	oleanolic acid	$C_{30}H_{48}O_{3}$	456.3603	OA
49	chikusetsusaponin IVa	$C_{42}H_{66}O_{14}$	794.4453	
50	ginsenoside Ro	$C_{48}H_{76}O_{19}$	956.4981	
51	chikusetsusaponin IV	$C_{47}H_{74}O_{18}$	926.4875	
52	pseudoginsenoside Rt1	$C_{47}H_{74}O_{18}$	926.4875	
53	24(*R*)-pseudoginsenoside Rt5	$C_{36}H_{62}O_{10}$	654.4343	OT
54	24(*R*)-pseudoginsenoside F11	$C_{42}H_{72}O_{14}$	800.4922	
55	ginsenoside Rk1	$C_{42}H_{70}O_{12}$	766.4868	Others
56	ginsenoside Rg5	$C_{42}H_{70}O_{12}$	766.4868	
57	5,6-didehydroginsenoside Rb1	$C_{54}H_{90}O_{23}$	1106.5873	
58	ginsenoside Rk3	$C_{36}H_{60}O_{8}$	620.4288	
59	ginsenoside Rh4	$C_{36}H_{60}O_{8}$	620.4288	
60	notoginsenoside T5	$C_{41}H_{68}O_{12}$	752.4711	

Compound I: 6-*O*-*β*-D-(6'-acetyl)-glucopyranosyl-24-en-dammar-3,6,12,20(*S*)-tetraol; Compound II: 6-*O*-(*β*-D-glucopyranosyl)-20-*O*-(*β*-D-xylopyranosyl)-3,6α,12,20(*S*)-tetrahydroxydammar-24-ene.

Table S2 Information of the 178 saponins identified in *Panax japonicus* by integrated analyses of the DDA and HDMS^E data.

No.	Observed RT (min)	Observed *m/z*	Formula	Mass error (ppm)	CCS (A^2)	Adducts	ESI-MS^2	Identification
1	2.35	861.4835	$C_{42}H_{72}O_{15}$	-2.2	305.45	+HCOO	815.5313,653.4333,654.4292,635.4110,553.3206,491.3715,415.3225,391.2855,389.2696,113.0251	(PPT+O)-Glc-Glc
2	2.50	863.4999[e]	$C_{42}H_{74}O_{15}$	-1.3	306.58	+HCOO	781.4889,655.4346,493.3906,417.3369,415.3210,391.2955,222.9747	(PPT+H_2O)-Glc-Glc
3	2.56	1007.5417[e]	$C_{48}H_{82}O_{19}$	-1.5	333.02	+HCOO	961.5174,799.4812,781.4759,653.4232,637.4103,635.4149,491.3750,415.3243,391.2851,373.2748,113.0248	OT-Glc-Glc-Rha
4	2.67	1023.5363	$C_{48}H_{82}O_{20}$	-1.8	320.03	+HCOO	977.5364,815.4852,653.4289,533.3832,491.3722,415.3218	OT-Glc-Glc-Glc
5	2.84	879.4952	$C_{42}H_{74}O_{16}$	-0.8	294.66	+HCOO	833.4940,671.4375,509.3848,391.2857,113.0249	(PPT+O+H_2O)-Glc-Glc
6	2.98	993.5266	$C_{47}H_{80}O_{19}$	-1.0	317.11	+HCOO,-H	947.5210,815.4776,653.4266,533.3784,491.3715,415.3214	OT-Xyl-Glc-Glc (Vinaginsenoside R6 or isomer)
7	3.48	1007.5432[e]	$C_{48}H_{82}O_{19}$	-0.1	322.82	+HCOO	961.5424,801.5027,799.4927,653.4297,491.3693,415.3238,403.3169,113.0246	OT-Glc-Rha-Glc
8	3.56	849.4849[b,e+]	$C_{41}H_{72}O_{15}$	0.5	292.5	+HCOO	803.4811,671.4395,509.3850,391.2861,113.0217	(PPT+H_2O+O)-Xyl-Glc
9	4.10	861.4870	$C_{42}H_{72}O_{15}$	1.9	295.6	+HCOO	815.4667,715.4003,653.4090,635.3881,553.3379,493.3866,491.3762,391.2855	(PPT+O)-Glc-Glc

10	4.53	1007.5427	$C_{48}H_{82}O_{19}$	-0.6	320.5	+HCOO	962.5414,961.5401,799.4857,637.4335,475.3803,391.2857,221.0657	PPT-Glc-(Glc-Glc)
11	4.72	861.4849	$C_{42}H_{72}O_{15}$	-0.5	296.03	+HCOO	815.4781,653.4304,491.3742,391.2856,389.2701,113.0234	(PPT+O)-Glc-Glc
12	4.85	1153.6002	$C_{54}H_{92}O_{23}$	-0.8	339.94	+HCOO	1107.5938,945.5401,799.4826,637.4345,475.3794,391.2865,221.0663	PPT-Glc-Rha-(Glc-Glc) (Yesanchinoside E or ginsenoside Re8 or isomer)
13	4.89	861.4853	$C_{42}H_{72}O_{15}$	-0.1	296.95	+HCOO	815.4814,653.4335,491.3751,391.2860,389.2701,113.0239	(PPT+O)-Glc-Glc
14	5.53	1007.5426[a]	$C_{48}H_{82}O_{19}$	-0.6	329.77	+HCOO,-H	961.5401,799.4860,637.4330,475.3801,475.3801,391.2859,389.2687,113.0246	20-*O*-Glucosyl-ginsenoside Rf
15	5.87	977.5311[a]	$C_{47}H_{80}O_{18}$	-1.6	317.45	+HCOO,-H	932.5561,768.5239,637.4389,553.3368,475.3780,391.2887,191.0559,131.0338	Notoginsenoside Fp1
16	5.98	831.4741	$C_{41}H_{70}O_{14}$	-0.8	290.01	+HCOO,-H	785.4603,653.4278,491.3743,391.2852	(PPT+O)-Xyl-Glc
17	6.10	977.5319[a]	$C_{47}H_{80}O_{18}$	-0.8	327.97	+HCOO	931.5364,799.4777,637.4313,475.3796,391.2860,113.0243	Notoginsenoside R1
18	6.18	861.4850[e]	$C_{42}H_{72}O_{15}$	-0.3	307.1	+HCOO	815.4804,653.4230,491.3771,415.3240,403.3229,161.0455,113.0245	OT-Glc-Glc
19	6.22	1123.5916	$C_{53}H_{90}O_{22}$	1.0	337.87	+HCOO,-H	1077.5867,945.5413,783.4976,637.4328,475.3801,391.2864,191.0561,131.0351	PPT-Xyl-Glc-Rha-Glc

20	6.26	1007.5439[a]	$C_{48}H_{82}O_{19}$	0.7	319.89	+HCOO	961.5399,799.4777,637.4376,475.3819,391.2868,113.0230	Ginsenoside Re2
21	7.32	845.4904[a]	$C_{42}H_{72}O_{14}$	0.0	301.66	+HCOO	799.4790,637.4331,475.3801,391.2857,389.2692,113.0247	Ginsenoside Rg1
22	7.63	991.5490[a]	$C_{48}H_{82}O_{18}$	0.7	332.03/261.05	+HCOO,-H	945.5420,783.4904,637.4330,475.3797,391.2856,389.2706,205.0710,113.0247	Ginsenoside Re
23	7.66	831.4749	$C_{41}H_{70}O_{14}$	0.2	300.07	+HCOO,-H	785.4769,653.4229,491.3719,415.3188	OT-Xyl-Glc
24	8.44	1195.6113	$C_{56}H_{94}O_{24}$	-0.4	345.6	+HCOO,-H	1149.6618,1107.6024,945.5489,783.4956,637.4345,475.3816,391.2862,161.0457	PPT-Glc-Glc-Rha-Glc-Ace.
25	8.59	1025.5537	$C_{48}H_{84}O_{20}$	-0.1	339.4	+HCOO	1025.5183,817.4938,799.4868,655.4445,637.4415,493.3900,375.2945,113.0251	(PPT+H_2O)-Glc-Glc-Glc
26	8.64	1005.5271	$C_{48}H_{80}O_{19}$	-0.4	324.21	+HCOO	959.5317,797.4761,635.4142,617.4080,473.3648,345.2431,331.2279,113.0255	(7-OH-5-ene-PPD)-Glc-Glc-Glc (Ginsenoside III or isomer)
27	8.72	843.4749	$C_{42}H_{70}O_{14}$	0.1	300.83	+HCOO	635.4195,636.4166,551.3117,473.3623,389.2692	(7-OH-5-ene-PPD)-Glc-Glc
28	8.83	887.5011[c]	$C_{44}H_{74}O_{15}$	0.1	310.72	+HCOO,-H	841.4970,637.4281,619.4206,535.3266,475.3789,457.3671,391.2851,	PPT-Ace.-Glc-Glc
29	8.95	1031.5420	$C_{51}H_{84}O_{21}$	-1.2	327.68	-H	945.5292,783.4976,637.4315,475.3787,391.2862,113.0231	PPT-Mal.-Glc-Rha-Glc
30	8.95	1005.5263	$C_{48}H_{80}O_{19}$	-1.2	336.62	+HCOO	959.5158,797.4652,635.4367,636.4347,473.3639,389.2709,381.3122,355.2677,113.0247	(7-OH-5-ene-PPD)-Glc-Glc-Glc (Notoginsenoside G or isomer)

31	9.04	831.4752[e]	$C_{41}H_{70}O_{14}$	0.5	289.62	+HCOO,-H	785.4732,653.4313,491.3717,415.3201,403.3209	OT-Xyl-Glc
32	9.11	1165.5997[e]	$C_{55}H_{92}O_{23}$	-1.2	343.64	+HCOO	1119.6060,1078.6024,1077.5510,945.5423,783.4891,637.4396,475.3796,391.2889,191.0562,131.0329	PPT-Ace.-Xyl-Glc-Rha-Glc
33	9.12	973.4999[b,e+]	$C_{48}H_{78}O_{20}$	-1.5	—	-H	973.4930,811.4465,749.4496,635.4390,677.4314,631.3837,587.3960,541.3920,473.3628,113.0268	(7-OH-5-ene-PPD)-Glc-GlurA-Glc
34	9.13	961.5387[e]	$C_{47}H_{80}O_{17}$	1.0	323.12	+HCOO,-H	915.5172,753.4710,607.4208,475.3805,391.2849	PPT-Glc-Rha-Xyl
35	9.23	1167.5826[c]	$C_{54}H_{90}O_{24}$	1.9	358.62	+HCOO,-H	852.3967,587.3933,475.3802,473.3634,391.2850,389.2656,	Notoginsenoside B or isomer
36	9.24	1031.5434[a]	$C_{51}H_{84}O_{21}$	0.2	—	-H	987.6656,945.5181,783.5231,637.4219,475.3829,391.2833,113.0234	Malonyl-floralginsenoside Re1
37	9.41	861.7562[b]	$C_{42}H_{72}O_{15}$	4.2	292.96	+HCOO	815.4833,653.4274,491.3767,415.3231,221.0668,161.0464,113.0247	OT-(Glc-Glc)
38	9.44	961.5397	$C_{47}H_{80}O_{17}$	2.0	—	-H,+HCOO	914.3988,915.3973,870.4121,783.4974,707.3456,637.4349,475.3842,391.2904,	PPT-Xyl-Rha-Glc
39	9.45	861.4859	$C_{42}H_{72}O_{15}$	0.7	309.2	+HCOO,-H	815.4819,653.4290,491.3757,415.3227,403.3225,401.3062,161.0461,113.0245	OT-Glc-Glc (Floralginsenoside B or isomer)

40	9.50	989.5317[e]	$C_{48}H_{80}O_{18}$	-0.9	330.73	+HCOO	943.5617,797.4885,780.4569,763.4728,635.4227,617.4048,475.3782,473.3555,455.3687,391.2916,345.2444,331.2373,276.2122,129.0211	(7-OH-5-ene-PPD)-Rha-Glc-Glc
41	9.53	1007.5436	$C_{48}H_{82}O_{19}$	0.4	335.1	+HCOO,-H	961.5503,799.4435,781.4798,619.4081,537.3487,475.4054,391.2888,375.2747,113.0251	PPT-Glc-Glc-Glc
42	9.56	887.5011[e]	$C_{44}H_{74}O_{15}$	0.1	307.03	+HCOO	686.2477,621.2611,620.4196,475.3785,391.2863	PPT-Glc-Glc-Ace.
43	9.72	1033.5591	$C_{50}H_{84}O_{19}$	0.2	339.22	-H	987.4163,945.5452,783.4920,637.4336,475.3797,391.2858,113.0244	PPT-Ace.-Glc-Rha-Glc
44	9.75	971.4860[d,e+]	$C_{48}H_{76}O_{20}$	0.3	322.47/249.56/201.06	-H	971.4892,809.4357,791.4247,521.3631,471.3516,451.3234,409.3473,113.0239	(OA+O)-2Glc-GlurA
45	9.93	977.5313	$C_{47}H_{80}O_{18}$	-1.4	324.56	+HCOO	748.4573,649.3142,475.3837,391.2831,353.1087,245.0592,221.0644,131.0378	PPT-Glc-Glc-Xyl
46	10.13	831.4754	$C_{41}H_{70}O_{14}$	0.7	301.33	+HCOO,-H	785.4709,653.4280,491.3758,415.3225,403.3227,161.0459,161.0459,113.0249	OT-Xyl-Glc
47	10.27	989.5337	$C_{48}H_{80}O_{18}$	1.0	332.62	+HCOO	943.5271,781.4818,619.4233,527.3795,457.3718,409.3564,221.0641,161.0455	Quinquenoside L_1 or isomer
48	10.47	1015.4756[b,e]	$C_{48}H_{74}O_{20}$	0.05	—	+HCOO	969.4722,807.4185,645.3678,627.3552,537.3592,469.3247,465.3387,423.3258	$(OA+CH_2)$-GlurA-Glc-Glc

49	10.53	845.4907[a]	$C_{42}H_{72}O_{14}$	0.3	304.52	+HCOO	799.4873,653.4303,491.3758,415.3224,161.0463,113.0248	24(*R*)-Pseudoginsenoside F11
50	10.71	955.4903[e]	$C_{48}H_{76}O_{19}$	-0.5	—	-H	955.4911,793.4391,631.3964,613.3760,569.3853,455.3533,437.3428,153.0195	OA-GlurA-Glc-Glc
51	10.97	1285.643[a]	$C_{59}H_{100}O_{27}$	-0.3	374.21	+HCOO	1239.6395,1107.5968,945.5447,783.4910,621.4372,459.3848,353.1092,221.0666,161.0456	Notoginsenoside R4
52	11.27	1117.5439[e]	$C_{54}H_{86}O_{24}$	0.2	355.99/258.50/217.27	-H	1117.5448,955.4835,793.4387,631.3845,569.3857,523.3806,455.3533,221.0673	OA-GlurA-Glc-Glc-Glc
53	11.50	845.4918[a]	$C_{42}H_{72}O_{14}$	1.7	311.53	+HCOO,-H	799.4871,637.4340,475.3803,391.2859,373.2750,113.0247	Ginsenoside Rf
54	11.56	971.4867[d,e+]	$C_{48}H_{76}O_{20}$	1	329.39	-H	971.4880,809.4388,747.4339,647.3989,629.3666,585.3810,471.3496,453.3412	(OA+O)-GlurA-Glc-Glc
55	11.62	1117.5458[e]	$C_{54}H_{86}O_{24}$	1.9	344.41	-H	1117.5447,955.4945,793.4380,731.4386,613.3742,569.3861,455.3533	OA-GlurA-Glc-Glc-Glc
56	11.75	1325.6393	$C_{62}H_{102}O_{30}$	0.7	379.14	-H	1281.6581,1239.6356,1107.5964,945.5417,783.4910,621.4377,459.3856,353.1094,221.0670,161.0458	PPD-Mal-Xyl-Glc-Glc-(Glc-Glc) (Malony-lginsenoside Ra3*)*
57	11.87	985.5013[d,e+]	$C_{49}H_{78}O_{20}$	-0.1	285.23	-H	985.5047,865.4668,823.4536,791.4221,646.3740,521.3663,472.3525,451.3220,409.3461,113.0255	(OA+O)-GlurA-Glc-GlurA

58	11.96	1137.6068	$C_{54}H_{92}O_{22}$	0.5	354.47	+HCOO	1091.6037,929.5448,767.4954,605.4447,565.7196,456.3505,161.0461,113.0226	(DD-II)-Glc-Glc-Glc-Glc
59	12.01	1327.6522[c]	$C_{61}H_{102}O_{28}$	-1.3	387.74	+HCOO,-H	1239.6338,1221.6223,621.4346,570.3890,459.3861,375.2902	PPD-Glc-Glc-Glc-Glc-Xyl-Ace
60	12.03	971.4877[d,e+]	$C_{48}H_{76}O_{20}$	2.0	—	-H	971.4907,809.4357,767.4491,647.3821,629.3717,477.3740, 471.3434, 407.3337, 337.0736	(OA+O)-GlurA-Glc-Glc
61	12.23	1087.5344[e]	$C_{53}H_{84}O_{23}$	1.2	351.34	-H	955.4969,793.4248,631.3762,569.3858,455.3543	OA-GlurA-Xyl-Glc-Glc
62	12.34	815.4812[a]	$C_{41}H_{70}O_{13}$	1.7	301.89	+HCOO,-H	769.4711,637.4334,475.3803,391.2861	20(*S*)-Notoginsenoside R2 or Ginsenoside F3
63	12.34	1087.5343[b,e]	$C_{53}H_{84}O_{23}$	1.1	351.34	-H	1087.5345,955.4630,793.4443,763.4300,631.3923,613.3757,569.3861,497.3661,455.3661,101.0285	OA-GlurA-Xyl-Glc-Glc
64	12.37	769.4761[a]	$C_{41}H_{70}O_{13}$	2.3	—	-H	769.4775,637.4369,475.3818,391.2874	20(*S*)-Notoginsenoside R2 or Ginsenoside F3
65	12.49	1153.6026[a]	$C_{54}H_{92}O_{23}$	1.2	357.7	+HCOO	1107.5968,945.5434,783.4906,621.4368,537.3449,459.3850,375.2910,221.0671	Ginsenoside Rb1
66	12.69	763.4283[e]	$C_{41}H_{64}O_{13}$	1.2	216.75	-H	763.4175,631.3940,600.3996,569.3882,497.3656,455.3544	OA-GlurA-Xyl
67	12.74	1087.5342[e]	$C_{53}H_{84}O_{23}$	1.0	341.25	-H	1087.5333,955.4714,793.4400,631.3850,613.3756,569.3857,497.3646,455.3547	OA-GlurA-Xyl-Glc-Glc
68	12.78	887.5021	$C_{44}H_{74}O_{15}$	1.2	320.36	+HCOO,-H	841.5033,799.4879,637.4329,475.3808,3	PPT-Ace.-Glc-Glc

							91.2865,389.2706,113.0240	
69	13.16	1327.654[c,e]	$C_{61}H_{102}O_{28}$	0	384.09	+HCOO,-H	1239.6290,1221.6179,783.4856,621.4396,459.3818,	PPD-Glc-Glc-Glc-Glc-Xyl-Ace.
70	13.24	969.4719[b,e]	$C_{48}H_{74}O_{20}$	1.9	326.56	-H	969.4767,807.4190,645.3644,627.3537,537.3596,469.3348,393.3167,391.3014,113.0220	(OA+CH_2)-GlurA-Glc-Glc
71	13.25	829.4964[a]	$C_{42}H_{72}O_{13}$	1.1	306.03	+HCOO,-H	783.4924,637.4346,475.3805,391.2858	Ginsenoside Rg2
72	13.28	843.4761	$C_{42}H_{70}O_{14}$	1.6	300.75	+HCOO	797.4691,635.4136,473.3634,443.3570,427.3229,389.3156,161.0462	(7-OH-5-ene-PPD)-Glc-Glc
73	13.34	683.4388[a]	$C_{36}H_{62}O_9$	1.8	281.32	+HCOO,-H	637.4492,475.3805,391.2867	20(*S*)-Ginsenoside Rh1
74	13.36	955.4924[e]	$C_{48}H_{76}O_{19}$	1.6	319.27	-H	955.4913,793.4261,631.3875,497.3666,455.3548	OA-GlurA-Glc-Glc
75	13.40	1117.5449[e]	$C_{54}H_{86}O_{24}$	1.2	346.66	-H	1117.5477,955.4955,793.4418,631.3931,569.3859,523.3820,455.3543,221.0677,113.0238	OA-GlurA-Glc-Glc-Glc
76	13.41	829.4970[e]	$C_{42}H_{72}O_{13}$	1.8	—	+HCOO,-H	783.5003,637.4266,475.3789,391.2886,201.1129	PPT-Rha-Glc
77	13.43	683.4393	$C_{36}H_{62}O_9$	2.5	—	+HCOO	476.3868,391.2801,	PPT-Glc
78	13.47	1193.5979[a]	$C_{57}H_{94}O_{26}$	1.5	355.39/266.72	-H	1149.6189,1107.6010,945.5456,783.4897,621.4383,459.3853,375.2907,221.0669,161.0460,113.0247	Malonyl-ginsenoside Rb1
79	13.50	683.4409	$C_{36}H_{62}O_9$	4.9	—	+HCOO	588.3666,475.3926,391.3037	PPT-Glc

80	13.57	1087.5347[e]	$C_{53}H_{84}O_{23}$	1.5	364.26	-H	1087.5506,955.4888,793.4437,731.4397,631.3785,569.3881,523.3821,455.3536,113.0232	OA-GlurA-Xyl-Glc-Glc
81	13.59	955.4921	$C_{48}H_{76}O_{19}$	1.4	329.58	-H	955.4935,793.4412,731.4392,659.4184,631.3882,613.3764,569.3869,455.3544	OA-GlurA-Glc-Glc
82	13.61	829.4970	$C_{42}H_{72}O_{13}$	1.8	309.46	+HCOO,-H	783.4966,637.4386,475.3804,391.2864,113.0267	PPT-Rha-Glc
83	13.63	1117.5454[e]	$C_{54}H_{86}O_{24}$	1.6	351.06	-H	1117.5493,955.4941,793.4422,631.3896,613.3767,569.3875,455.3547,113.0249	OA-GlurA-Glc-Glc-Glc
84	13.84	683.4387[a]	$C_{36}H_{62}O_{9}$	1.6	286	+HCOO,-H	637.4387,475.3763,391.2851	20(*R*)-Ginsenoside Rh1
85	13.87	1195.6144[e]	$C_{56}H_{94}O_{24}$	2.3	372.49	+HCOO	1149.6028,1107.6006,945.5451,783.4878,621.4363,459.3847,375.2909,221.0666,113.0237	PPD-Ace.-Glc-Glc-Glc-Glc
86	13.91	989.5342	$C_{48}H_{80}O_{18}$	1.6	330.29	+HCOO	943.5373,781.4804,731.4421,619.4149,457.3692,377.2951,113.0250	(Dehydrated-PPT)-Glc-Glc-Glc
87	13.91	955.4910[b,e]	$C_{48}H_{76}O_{19}$	0.2	252.25	-H	955.4973,793.4392,731.4414,631.3832,569.3875,523.3811,455.3540,113.0248	OA-GlurA-Glc-Glc
88	13.96	887.5021[e]	$C_{44}H_{74}O_{15}$	1.3	312.63	+HCOO	841.4936,799.4854,653.4262,637.4458,619.4069,491.3802,475.3785,415.3247,391.2878,331.2203,161.0467	PPT-Ace.-Glc-Glc
89	14.12	1123.5913[a]	$C_{54}H_{92}O_{24}$	0.7	368.79	+HCOO,-H	1077.5746,945.5028,783.4871,621.4244,459.3879,191.0590	Ginsenoside Rb2

90	14.23	955.4918[e]	$C_{48}H_{76}O_{19}$	1.0	334.01/260.21	-H	955.4961,793.4409,749.4517,731.4400,631.3892,613.3758,569.3859,455.3561,409.3489,113.0253	OA-GlurA-Glc-Glc
91	14.37	1087.5332[e]	$C_{53}H_{84}O_{23}$	0.1	368.05/277.04/220.63	-H	1087.5372,955.4902,793.4421,731.4384,631.3824,631.3824,569.3853,455.3534,113.0242	OA-GlurA-Xyl-Glc-Glc
92	14.40	1117.5434[e]	$C_{54}H_{86}O_{24}$	-0.2	352.16	-H	1117.5387,955.4923,793.4240,731.4370,713.4285,631.3866,613.3762,569.3862,523.3794,455.3528,221.0663,113.0234	OA-GlurA-Glc-Glc-Glc
93	14.46	1123.5902[a]	$C_{53}H_{90}O_{22}$	-0.3	360.52	+HCOO,-H	1077.5861,945.5433,783.4920,621.4389,459.3844,375.2913,191.0565,113.0249	Ginsenoside Rb3
94	14.58	1057.5227	$C_{52}H_{82}O_{22}$	0.2	346.61	-H	1057.5292,925.4803,763.4247,631.3854,613.3757,569.3854,497.3646,455.3539,113.0253	OA-GlurA-Xyl-Glc-Xyl-
95	14.83	955.4909[a]	$C_{48}H_{76}O_{19}$	0.1	329.02/254.24	-H	955.4905,793.4373,569.3846,455.3529	Ginsenoside Ro
96	14.83	1087.5334[b,e]	$C_{53}H_{84}O_{23}$	0.3	365.74	-H	1087.5457,955.4876,793.4329,631.3825,613.3790,569.3876,569.3876,455.3529,113.0220	OA-GlurA-Xyl-Glc-Glc
97	14.86	1123.5914	$C_{53}H_{90}O_{22}$	0.7	345.16	+HCOO	1077.5658,945.5368,783.4875,621.4241,459.3777,353.1093,221.0660,131.0353	PPD-Xyl-Glc-Glc-Glc
98	14.86	683.4386[a]	$C_{36}H_{62}O_{9}$	1.5	279.17	+HCOO	475.3727,391.2890	Ginsenoside F1
99	15.20	1087.5337	$C_{53}H_{84}O_{23}$	0.6	369.22/266.58/219.86	-H	1087.5345,925.4816,793.4397,763.4287,731.4377,631.3871,569.3853,551.3746,4	OA-GlurA-Glc-Xyl-Glc

							55.3534,113.0247	
100	15.23	955.4917	$C_{48}H_{76}O_{19}$	0.9	328.57	-H	955.4917,793.4395,731.4379,631.3862,613.3748,569.3853,455.3533	OA-GlurA-Glc-Glc
101	15.35	725.4490[c]	$C_{38}H_{64}O_{10}$	1.2	288.84	+HCOO,-H	661.4249,555.3654,475.3783,401.2676	PPT-Glc-Ace.
102	15.36	925.4810[e]	$C_{47}H_{74}O_{18}$	0.9	—	-H	925.4819,793.4446,631.3861,569.3852,455.3537,113.0266	OA-GlurA-Xyl-Glc
103	15.37	1195.6141	$C_{56}H_{94}O_{24}$	2.0	363.72	+HCOO	1150.6208,1149.5755,1107.6069,1089.5915,945.5308,783.4867,621.4402,459.3865,375.2923,221.0678	PPD-Ace.-Glc-Glc-Glc-Glc
104	15.40	1087.5331[b,e]	$C_{53}H_{84}O_{23}$	0.04	—	-H	1087.5277,955.4698,793.4313,631.3861,569.3811,551.3762,455.3522,221.0724	OA-GlurA-Xyl-Glc-Glc
105	15.49	1057.5226[e]	$C_{52}H_{82}O_{22}$	0.1	358.15	-H	1057.5283,925.4730,793.4351,631.4031,523.3814,455.3534,113.0252	OA-GlurA-Xyl-Xyl-Glc
106	15.52	955.4909[e]	$C_{48}H_{76}O_{19}$	0.1	259.96	-H	955.4916,793.4384,631.3859,613.3745,569.3856,455.3534,113.0251	OA-GlurA-Glc-Glc
107	15.58	1163.5849	$C_{56}H_{92}O_{25}$	-0.5	356.92	-H	1119.6012,1077.5905,945.5446,783.4911,621.4356,459.3803,375.2923,113.0248	PPD-Mal.-Glc-Ara-Glc-Glc
108	15.60	925.4804[e]	$C_{47}H_{74}O_{18}$	0.2	—	-H	925.4957,793.4412,631.3911,613.3753,569.3859,497.3636,455.3542	OA-GlurA-Xyl-Glc

109	15.74	955.4901[e]	$C_{48}H_{76}O_{19}$	-0.8	337.3	-H	955.4914,793.4381,731.4409,631.3839,613.3750,569.3863,523.3793,455.3546,113.0240	OA-GlurA-Glc-Glc
110	15.85	925.4798[e]	$C_{47}H_{74}O_{18}$	-0.5	—	-H	925.4807,763.4270,631.4151,613.3747,569.3854,455.3531,113.0247	OA-GlurA-Glc-Xyl
111	15.85	955.4902[b,e]	$C_{48}H_{76}O_{19}$	-0.6	—	-H	955.4944,793.4398,731.4355,631.3731,613.3753,569.3852,523.3817,455.3525	OA GlurA-Glc-Glc-
112	15.94	939.4953	$C_{48}H_{76}O_{18}$	-0.6	327.02/253.05/205.44	-H	925.4712,763.4339,631.3818,613.3755,569.3853,497.3644,455.3547	OA-GlurA-Xyl-Glc
113	15.94	1057.5229[e]	$C_{52}H_{82}O_{22}$	0.4	355.59	-H,+HCOO	1057.5293,895.4679,763.4443,701.4243,601.5267,587.3937,569.3869,455.3514,113.0249	OA-GlurA-Glc-Xyl -Xyl
114	15.94	925.4807[b,e]	$C_{47}H_{74}O_{18}$	0.5	—	-H	925.4808,793.4274,763.4253,701.4258,631.3937,613.3754,569.3847,455.3539	OA-GlurA-Xyl-Glc
115	15.94	955.4905[e]	$C_{48}H_{76}O_{19}$	-0.3	—	-H	955.4922,793.4413,731.4374,631.3832,613.3756,569.3865,523.3808,455.3529,113.0251	OA-GlurA-Glc-Glc
116	16.09	991.5467[a]	$C_{48}H_{82}O_{18}$	-1.6	336.69	+HCOO,-H	945.5457,783.4865,621.4379,537.3429,459.3841,375.2902,373.2750,221.0666,113.0243	Ginsenoside Rd

117	16.16	941.5097	$C_{48}H_{78}O_{18}$	-1.9	335.24	-H	941.5074,793.4395,779.4754,731.4376,615.3722,613.3815,569.3825,455.3712,439.3663,113.0240	OA-Glc-Glc-Glc
118	16.18	955.4894[e]	$C_{48}H_{76}O_{19}$	-1.5	253.87	-H	955.4893,793.4390,731.4377,631.3857,659.4163,613.3748,569.3847,523.3802,455.3530,113.0241	OA-GlurA-Glc-Glc
119	16.36	925.4808[c]	$C_{47}H_{74}O_{18}$	0.6	325.17/251.25	-H	925.4800,763.4270,613.3741,497.3634,455.3528	OA-GlurA-Xyl-Glc
120	16.38	939.4938	$C_{48}H_{76}O_{18}$	-2.2	327.27	-H	939.4965,777.4427,715.4431,631.3789,613.3751,569.3849,455.3520,113.0265	OA-GlurA-Glc-Rha
121	16.41	793.4377[e]	$C_{42}H_{66}O_{14}$	-0.3	—	-H	793.4432,631.3871,569.3877,455.3527,113.0227	OA-GlurA-Glc
122	16.57	925.4791[a]	$C_{47}H_{74}O_{18}$	-1.2	—	-H	925.4800,763.4271,569.3844,455.3527,113.0244	Pseudoginsenoside Rt1
123	16.77	925.4803[c]	$C_{47}H_{74}O_{18}$	-0.1	329.71/249.93	-H	925.4789,793.4376,613.3738,497.3634,455.3529,	OA-GlurA-Xyl-Glc
124	16.90	925.4803[c,e]	$C_{47}H_{74}O_{18}$	-0.1	—	-H	925.4789,731.4357,613.3738,497.3633,455.3528,	OA-GlurA-Xyl-Glc
125	17.07	1041.4899[b,e+]	$C_{51}H_{78}O_{22}$	-1.3	—	-H	997.4990,835.4502,659.4149,569.3848,497.3632,455.3535	OA-GlurA-Mal-Glc-Glc
126	17.12	997.5006[b,e+]	$C_{50}H_{78}O_{20}$	-0.8	—	-H	997.4991,835.4495,659.4238,569.3867,497.3636,455.3541,113.0275	OA-GlurA-Ace.-Glc -Glc

127	17.26	925.4793[a]	$C_{47}H_{74}O_{18}$	-1.0	330.41	-H	925.4809,763.4269,569.3844,497.3637,455.3531	Chikusetsusaponin IV
128	17.39	1033.5561[c]	$C_{50}H_{84}O_{19}$	-2.7	346.21	+HCOO,-H	945.5412,783.4860,621.4367,538.3502,441.3727,376.2931	PPD-3Glc-Ace.
129	17.41	1031.543[a]	$C_{51}H_{84}O_{21}$	-0.2	344.4	-H	987.5768,945.5436,783.4899,621.4377,459.3846,375.2913,161.0448,113.0249	Malonyl-ginsenoside Rd
130	17.51	1031.5407	$C_{51}H_{84}O_{21}$	-2.4	—	-H	998.4588,988.5671,946.5313,621.4335,569.3801,459.3676,375.2884,113.0242	PPD-Mal.-Glc-Glc-Glc
131	17.51	997.5000[b,e+]	$C_{50}H_{78}O_{20}$	-1.4	247.46	-H	997.5015,835.4480,659.4171,613.3736,569.3842,497.3646,455.3530,113.0235	OA-GlurA -Ace.-Glc-Glc
132	17.56	939.4946	$C_{48}H_{76}O_{18}$	-1.3	322.1	-H	939.4987,777.4439,715.4427,643.4202,631.3714,613.3742,569.3841,455.3530,113.0256	OA-GlurA-Glc-Rha
133	17.57	991.5471[a]	$C_{48}H_{82}O_{18}$	-1.3	327.85	+HCOO	945.5342,783.4998,621.4371,459.3684,375.2955	Notoginsenoside K
134	17.66	925.479[e]	$C_{47}H_{74}O_{18}$	-1.3	—	-H	925.4676,793.4386,631.3854,613.3725,569.3843,497.3634,455.3531	OA-GlurA-Xyl-Glc
135	17.84	793.4371[a]	$C_{42}H_{66}O_{14}$	-1.2	289.49/223.81	-H	793.4368,631.3841,569.3841,455.3524	Chikusetsusaponin IVa

136	18.14	1031.5423	$C_{51}H_{84}O_{21}$	-0.9	327.05	-H	987.5468,945.5412,783.4890,621.4376,459.3836,375.2891,161.0476,113.0244	PPD-Mal.-Glc-Glc-Glc
137	18.16	997.5019[b,e+]	$C_{50}H_{78}O_{20}$	0.53	—	-H	997.5147,835.4419,659.4222,569.3858,497.3631,455.3509,113.0240	OA-GlurA-Ace.-Glc -Glc
138	18.21	871.5069[c]	$C_{44}H_{74}O_{14}$	1.0	313.57	+HCOO	614.3776,527.3748,475.3792,391.2855	PPT-Glc-Rha-Ace.
139	18.42	997.5003[b,e+]	$C_{50}H_{78}O_{20}$	-1.1	338.39	-H	997.4951,835.4479,659.4148,569.3856,497.3647	OA-GlurA-Ace.-Glc -Glc
140	18.48	793.4374	$C_{42}H_{66}O_{14}$	-0.8	292.09	-H	793.4378,631.3850,569.3847,497.3641,455.3530,113.0248	OA-GlurA-Glc
141	18.83	997.4996[b,e+]	$C_{50}H_{78}O_{20}$	-1.8	338.39	-H	997.5023,835.4476,659.4172,497.3653,455.3530,113.0249	OA-GlurA-Ace.-Glc -Glc
142	18.88	925.4800[b,e]	$C_{47}H_{74}O_{18}$	-0.3	337.6	-H	925.4789,793.4395,631.3994,613.3799,569.3850,497.3646,455.3532,113.0255	OA-GlurA-Xyl-Glc
143	18.96	793.4380[e]	$C_{42}H_{66}O_{14}$	0.1	—	-H	793.4380,631.3857,569.3858,497.3636,455.3545,113.0241	OA-GlurA-Glc
144	19.12	793.4376[e]	$C_{42}H_{66}O_{14}$	-0.5	293.84	-H	793.4348,631.3850,569.3846,455.3536,113.0258	OA-GlurA-Glc

145	19.19	827.4797	$C_{42}H_{70}O_{13}$	-0.2	316.1	+HCOO,-H	781.4487,619.4473,457.3882,101.0219	(Dehydrated-PPT)-Glc-Glc
146	19.23	1031.5427	$C_{51}H_{84}O_{21}$	-0.5	341.37	-H	945.5580,783.4825,621.4403,459.3834,375.2915,113.0248	PPD-Mal.-Glc-Glc-Glc
147	19.24	1117.5434	$C_{54}H_{86}O_{24}$	-0.2	339.93	-H	998.4415,945.5410,927.5246,783.5043,621.4355,569.3769,459.3866,375.2889,113.0203	PPD-Mal.-Mal.-Glc-Glc-Glc (Malonyl-floralginsenoside Rd6 or isomer)
148	19.26	1015.5120	$C_{49}H_{78}O_{19}$	0.1	330.16	+HCOO	969.3931,807.4578,627.3911,537.3590,455.3539,153.0191	Chikusetsusaponin V methyl ester or isomer
149	19.27	961.5386	$C_{47}H_{80}O_{17}$	0.9	328.02	+HCOO,-H	915.5304,783.4933,621.4372,537.3540,459.3824,375.2924,131.0346	PPD-Xyl-Glc-Glc
150	19.30	1033.5571	$C_{50}H_{84}O_{19}$	-1.7	342.49	+HCOO	987.5504,945.5420,783.4794,621.4328,459.3841,375.2892,161.0453	PPD-Ace.-Glc-Glc-Glc
151	19.35	997.5008[b,e+]	$C_{50}H_{78}O_{20}$	-0.6	338.39	-H	997.5018,835.4514,659.4182,569.3847,497.3647,455.3532,113.0247	OA-GlurA-Ace.-Glc -Glc
152	19.35	1031.5420	$C_{51}H_{84}O_{21}$	-1.2	343.01	-H	987.5566,945.5679,783.4898,621.4122,459.3883,375.2875	PPD-Mal.-Glc-Glc-Glc
153	19.36	1129.5437[b,e+]	$C_{55}H_{86}O_{24}$	0.1	391.04	-H	1129.5465,997.5039,835.4324,659.4204,497.3639,455.3528,113.0260,	OA-GlurA-Ace.-Xyl-Glc -Glc
154	19.38	763.4276[e]	$C_{41}H_{64}O_{13}$	0.3	—	-H	763.3987,587.4076,569.3833,497.3655,455.3561	OA-GlurA-Xyl

155	19.43	939.4929	$C_{48}H_{76}O_{18}$	-3.2	350.02	-H	939.4892,793.4322,631.3816,613.3766,569.3874,455.3499	OA-GlurA-Rha-Glc
156	19.50	1029.5273[e]	$C_{50}H_{80}O_{19}$	-0.2	334.77	+HCOO	983.4504,821.4717,641.4058,537.3583,455.3527,437.3423	Chikusetsusaponin V ethyl ester or isomer
157	19.55	811.4843	$C_{42}H_{70}O_{12}$	-0.8	309.85	+HCOO,-H	765.4634,619.4137,527.3779,457.3667,437.3394,113.0248	(Dehydrated-PPT)-Rha-Glc
158	19.6	853.4593	$C_{43}H_{68}O_{14}$	0.2	325.32	+HCOO	645.3990,569.3922,455.3578	Chikusetsusaponin IVa methyl ester or spinasaponinA methyl ester
159	19.79	829.4950[a]	$C_{42}H_{72}O_{13}$	-0.6	308.54	+HCOO,-H	783.4881,621.4439,537.3477,459.3847,375.2895	20(*S*)-Ginsenoside Rg3 or 20(*R*)-Ginsenoside Rg3
160	19.87	853.4946	$C_{44}H_{72}O_{13}$	-1.0	314.01	+HCOO	807.4884,765.4798,619.3965,457.3638	Ginsenoside Rs5 or isomer
161	19.98	811.4846	$C_{42}H_{70}O_{12}$	-0.4	315.84	+HCOO	765.4807,603.4317,439.3624,216.9875,113.0246	Isomer of ginsenoside Rg5
162	20.03	925.4798[e]	$C_{47}H_{74}O_{18}$	-0.5	—	-H	925.4782,793.4405,631.3873,613.3738,569.3855,551.3747,455.3531,113.0238	OA-GlurA-Xyl-Glc
163	20.07	955.4904[e]	$C_{48}H_{76}O_{19}$	-0.4	—	-H	955.4923,793.4389,659.4178,631.3881,613.3739,569.3846,523.3796,455.3532	OA-GlurA-Glc-Glc
164	20.08	925.4801[b,e]	$C_{47}H_{74}O_{18}$	-0.2	—	-H	925.4955,793.4084,631.3908,569.3843,497.3636,455.3531	OA-GlurA-Xyl-Glc

165	20.09	1261.6722	$C_{65}H_{100}O_{21}$	-1.4	377.54	+HCOO	1153.7508,1037.6027,955.4882,793.4400,613.3733,569.3845,455.3536,317.1719,113.0262	Polyacetyleneginsenoside Ro
166	20.09	1231.6626	$C_{64}H_{98}O_{20}$	-0.6	363.04	+HCOO	1177.1316,925.4805,753.4965,613.3749,569.3847,497.3639,455.3539	Baisanqisaponin B
167	20.15	793.4382[e]	$C_{42}H_{66}O_{14}$	0.3	310.56	-H	793.4374,631.3866,613.3747,569.3854,453.3376,455.3525,437.3434,113.0244	OA-GlurA-Glc
168	20.24	811.4844[a]	$C_{42}H_{70}O_{12}$	-0.6	317.05	+HCOO,-H	765.4790,603.4299,412.9850,279.2314,161.0458,101.0258	Ginsenoside Rg5
169	20.29	925.4804[e]	$C_{47}H_{74}O_{18}$	0.2	344.65	-H	925.4922,793.4500,631.3925,613.3739,569.3862,455.3378,453.3378	OA-GlurA-Xyl-Glc
170	20.34	853.4938	$C_{44}H_{72}O_{13}$	-2.0	323.98	+HCOO	807.4909,765.4792,747.4735,603.4592,455.3496	Chikusetsusaponin IVa methyl ester or isomer
171	20.35	663.4117	$C_{36}H_{58}O_{8}$	0.5	274.01	+HCOO	617.0089,587.0176,455.3535	OA-Glc
172	20.46	763.4285[e]	$C_{41}H_{64}O_{13}$	1.4	—	-H	763.4204,631.3832,613.3760,569.3866,455.3504	OA-GlurA-Xyl
173	20.72	925.4805[e]	$C_{47}H_{74}O_{18}$	0.3	312.93	-H	925.4961,793.4306,731.4385,569.3848,551.3742,455.3530	OA-GlurA-Xyl-Glc
174	20.86	777.4445	$C_{42}H_{66}O_{13}$	1.9	309.79	-H	777.4525,616.4114,507.3856,437.3401	Bipinoside A or isomer

175	20.99	763.4279	$C_{41}H_{64}O_{13}$	0.6	313.25	-H	763.4530,631.3870,569.3853,497.3645,455.3535,116.9291	OA-GlurA-Xyl
176	21.12	631.3855[b,e+]	$C_{36}H_{56}O_{9}$	0.5	275.40	-H	631.3859,455.3536	OA-GlurA
177	21.28	763.4281[e]	$C_{41}H_{64}O_{13}$	0.9	—	-H	764.4292,569.3854,497.3651,455.3534	OA-GlurA-Xyl
178	24.23	455.3540[b,e]	$C_{30}H_{48}O_{3}$	2.0	228.32	-H	455.3536,407.3332,240.9894	Oleanolic acid

[a]: components identified by comparison with the reference compounds;

[b]: components identified in the “Unknown Components” based on the DDA data;

[c]: components characterized based on the $HDMS^E$ data;

[d]: components with unknown masses, but listed in the “Identified Components” based on the DDA data due to being identified as the FA-adducts of known components;

[e]: the compounds that may have not been isolated from the *Panax* genus;

[e+]: the compounds with unknown masses that are not included in the in-house database.
